# Health impacts of air pollution exposure from 1990 to 2019 in 43 European countries

**DOI:** 10.1038/s41598-021-01802-5

**Published:** 2021-11-18

**Authors:** Alen Juginović, Miro Vuković, Ivan Aranza, Valentina Biloš

**Affiliations:** 1 Department of Neurobiology, Harvard Medical School, 220 Longwood Avenue, Boston, MA 02115 USA; 2 School of Medicine, University of Split, Šoltanska 2, 21 000 Split, Croatia

**Keywords:** Climate change, Cancer epidemiology, Stroke, Myocardial infarction, Epidemiology, Risk factors, Lung cancer

## Abstract

Air pollution is the fourth greatest overall risk factor for human health. Despite declining levels in Europe, air pollution still represents a major health and economic burden. We collected data from the Global Burden of Disease Study 2019 regarding overall, as well as ischemic heart disease (IHD), stroke, and tracheal, bronchus and lung cancer-specific disability adjusted life years (DALYs), years of life lost (YLL) and mortality attributable to air pollution for 43 European countries between 1990 and 2019. Concentrations of ambient particulate matter (aPM_2.5_), ozone, and household air pollution from solid fuels were obtained from State of Global Air 2020. We analysed changes in air pollution parameters, as well as DALYs, YLL, and mortality related to air pollution, also taking into account gross national income (GNI) and socio-demographic index (SDI). Using a novel calculation, aPM_2.5_ ratio (PMR) change and DALY rate ratio (DARR) change were used to assess each country’s ability to decrease its aPM_2.5_ pollution and DALYs to at least the extent of the European median decrease within the analysed period. Finally, we created a multiple regression model for reliably predicting YLL using aPM_2.5_ and household air pollution. The average annual population-weighted aPM_2.5_ exposure in Europe in 1990 was 20.8 μg/m^3^ (95% confidence interval (CI) 18.3–23.2), while in 2019 it was 33.7% lower at 13.8 μg/m^3^ (95% CI 12.0–15.6). There were in total 368 006 estimated deaths in Europe in 2019 attributable to air pollution, a 42.4% decrease compared to 639 052 in 1990. The majority (90.4%) of all deaths were associated with aPM_2.5_. IHD was the primary cause of death making up 44.6% of all deaths attributable to air pollution. The age-standardised DALY rate and YLL rate attributable to air pollution were more than 60% lower in 2019 compared to 1990. There was a strong positive correlation (r = 0.911) between YLL rate and aPM_2.5_ pollution in 2019 in Europe. Our multiple regression model predicts that for 10% increase in aPM_2.5_, YLL increases by 16.7%. Furthermore, 26 of 43 European countries had a positive DARR change. 31 of 43 European countries had a negative PMR change, thus not keeping up with the European median aPM_2.5_ concentration decrease. When categorising countries by SDI and GNI, countries in the higher brackets had significantly lower aPM_2.5_ concentration and DALY rate for IHD and stroke. Overall, air pollution levels, air pollution-related morbidity and mortality have decreased considerably in Europe in the last three decades. However, with the growing European population, air pollution remains an important public health and economic issue. Policies targeting air pollution reduction should continue to be strongly enforced to further reduce one of the greatest risk factors for human health.

## Introduction

Clean air is considered one of the basic requirements of human health and well-being. However, more than 90% of the world population was exposed to air quality levels that exceeded the World Health Organization (WHO) Air Quality Guideline (AQG) limits in 2016^1^. Air pollution is the fourth greatest overall risk factor for human health globally, following high blood pressure, dietary risks, and smoking^2^. It has been associated with three of the leading causes of death in the world with significant shares of air pollution-related mortality: stroke (26%), ischemic heart disease (IHD) (20.2%), and primary cancer of the trachea, bronchus, and lung (TBL) (19%)^3,4^. Altogether, air pollution was linked to seven million deaths globally and in excess of 100 million disability-adjusted life years (DALYs) annually^5,6^. It also represents a major global annual economic impact of $5 trillion^7^.

Disparities in air quality have been observed based on a country's income. While air pollution in developed countries poses an important public health issue, it is even more pronounced in developing countries where fast-growing population along with widespread industrialization led to centers with poor air quality which became a serious threat to health^8^.

In Europe, emissions of air pollutants have been declining in the past decades^9^. Nevertheless, there were still more than 0.5 million deaths attributable to air pollution in 2013 while health-related external costs associated with air pollution reached close to €1 trillion annually in the European Union (EU) alone^10,11^.

In 2018, 73.6% of the EU urban population was exposed to excessive concentrations of particulate matter of diameter less than 2.5 microns (PM_2.5_) which is considered the fifth leading mortality risk factor. The main contributors to the EU's PM_2.5_ concentrations have been institutional, commercial, and household (55.5%), followed by road transport (10.7%)^12^. PM_2.5_ pollution was associated with more than four million global deaths in 2016 with Europe counting for approximately 10% of that share^13^. Exposure to PM_2.5_ pollution led to more than 1277 years of life lost (YLL) per 100,000 population in several European countries^14^. Long-term exposure to PM_2.5_ pollution significantly increases both cardiopulmonary problems and lung cancer mortality, as well as risk for type two diabetes^15,16^. Conversely, one study showed that patients with lung cancer increased their life span by 0.35 years for every 10 μg/m^3^ reduction of PM_2.5_ concentration^17^. For short term exposure, every 10 μg/m^3^ increase in PM_2.5_ concentration was associated with 2.8% increase in PM-related mortality^18^.

Another major air pollutant is ozone, commonly found in urban areas which make up as much as 74.7% of the total EU population in 2019^19^. Long-term exposure to ozone has been linked to an increased risk of death from respiratory causes, as well as serious adverse pregnancy outcomes^20,21^.

The Global Burden of Diseases, Injuries, and Risk Factors Study (GBD) is a multinational collaborative research study of disease burden that assesses mortality and disability from major diseases, injuries and risk factors, including air pollution^22^. The study is an ongoing effort and is designed to systematically incorporate information over time, and its latest iteration includes data from 1990 to 2019, by age and sex, and across more than 200 countries and territories. The study contains standard epidemiological measures such as incidence, prevalence, and death rates, as well as summary measures of health, such as DALYs.

Using the most recent 2019 GBD data, we did a comprehensive analysis of temporal and spatial trends for PM_2.5_ and ozone concentrations in Europe from 1990 to 2019 with a focus on ambient PM_2.5_ (aPM_2.5_) concentrations due to its highest health impact. We also evaluated each country's ability to decrease its aPM_2.5_ and DALY values to at least the extent of the European aPM_2.5_ and DALY median reduction. Then, we analysed mortality, DALYs, and YLL attributable to air pollution for stroke, IHD, and TBL cancer, also taking into account socio-demographic index (SDI) and gross national income (GNI). This analysis could be used to raise awareness among policymakers to take action on this important public health issue which originates mainly from anthropogenic sources, and as such can be undone by precise and determined measures.

## Methods

### Sources of data

Data for 43 European countries regarding overall mortality, YLL, and DALYs attributable to air pollution by age, year, and sex were collected using the Global Health Data Exchange GBD Results Tool between 1990 and 2019. We also collected data for stroke, IHD and TBL cancer attributable to aPM_2.5_, household air pollution from solid fuels, and ozone. Detailed description of metrics, data sources, and statistics in GBD 2019 has been reported elsewhere^22^.

Estimates for exposure levels to aPM_2.5_ and ozone were obtained from Health Effects Institute–State of Global Air 2020 where methods for assessing their exposure levels are also described^23^. In brief, exposure to aPM_2.5_ was measured as the average annual PM_2.5_ concentration in the air at a spatial resolution of a 0.1° × 0.1° grid cell, which reflects to 11 × 11 km at the equator. Ozone concentration is measured in parts per billion (ppb). Exposure to ozone was defined as the highest seasonal (six-month) average daily eight-hour maximum ozone concentration.

We used GBD 2019 estimations of deaths, YLLs, and DALYs attributable to air pollution. Relative risks were estimated based on meta-regression and systematic reviews done for GBD 2019. DALYs, YLLs, and attributable deaths were estimated by multiplying population attributable fractions (PAFs) by the relevant outcome quantity for each age, sex, location, and year. For continuous risk, PAFs are calculated using formula described in GBD 2019 study^22^. Inputs to estimation of PAFs for this study included continuous exposure distributions to air pollution, relative risk and the theoretical minimum risk exposure level (TMREL) for each group. TMREL was defined as the low point of the risk function and it represents the level of risk exposure that minimizes disease risk at the population level. Using PAF estimates, we calculated the number of deaths attributable to air pollution, DALYs and YLLs.

Data for SDI in 2019 for 43 European countries was obtained from the Global Burden of Disease Study 2019. For a robust analysis of disparities between groups in terms of aPM_2.5_ pollution and disease burden, we divided these countries into 3 groups: high (> 0.850), medium (0.750–0.849), low (< 0.749) (Supplementary Table S4).

GNI per capita for 39 out of 43 European countries was obtained from the 2019 World Bank classification of world economies. Data for Andorra, Moldova, Monaco, and San Marino were not available^24^. The World Bank assigns the world’s economies into four income groups: low (< $1036), lower-middle ($1036–$4045), upper-middle ($4046–$12,535), and high-income (> $12,536) countries. Since 30 out of 39 analysed European countries are classified as high income, we divided those countries into an additional three groups: lower high income ($12,536–$36,766), moderate high income ($36,767–$60,997), very high income (> $60,997) for more robust analysis of disparities in aPM_2.5_ pollution and disease burden between countries with different GNI (Supplementary Table S4). Also, since Ukraine is the only country in Europe classified as lower-middle income, we merged the lower-middle income and upper-middle income groups into one due to the same reasons.

### Definitions

DALYs represent the overall number of years of potential life lost due to premature mortality and years of productive life lost due to disability and it is calculated as the sum of the aforementioned parameters. It summarizes the overall burden of disease and one DALY may be regarded as one year of healthy life lost^25^. DALYs can be expressed as the number of total DALYs or as DALY rate per 100,000 population. Additional methodologies for estimating DALYs have been described as part of GBD Study 2019^22^.

YLL is regarded as a summary measure of premature mortality. It estimates the years of potential life lost due to premature death, taking into account frequency of deaths and the age at which it occurs. YLLs were calculated by multiplying the estimated number of deaths by age with a standard life expectancy at that age. It can be expressed as a number of total YLLs or as YLL rate per 100,000 population. Additional methodologies for estimating YLLs are described in the GBD Study 2019^22^.

Death can be expressed as the rate per 100,000 population or as the total number of deaths. To calculate deaths attributable to air pollution, the total number of deaths is multiplied by the population attributable fraction (PAF), which may be interpreted as the proportion of deaths attributable to air pollution. Additional methodologies for estimating the number of deaths are described in the GBD Study 2019^22^.

SDI is a summary measure of socio‐demographic development status, strongly correlated with health outcomes. It is a geometric mean of the rankings of the lag-distributed income per capita, mean educational attainment for those age 15 or older, and fertility rate in those under 25 years old. It is expressed on a scale of 0 to 1, but for GBD 2019, values were multiplied by 100 for a scale of 0 to 100, where a location with an SDI of 0 has a theoretical minimum level of SDI, and a location with an SDI of 1 (prior to multiplying by 100 for reporting purposes) would have a theoretical maximum level of sociodemographic development relevant to health outcomes. Additional descriptions about SDI calculation can be found in the GBD Study 2019^22^.

GNI per capita represents the value produced by a country’s economy in a given year per one person, regardless of whether the value is produced domestically or abroad. Methodologies for calculation of GNI per capita in U.S. dollars are based on the Atlas method exchange rates described elsewhere^26^.

DALY rate ratio (DARR) represents the ratio between a country's DALY rate for a given year and a median DALY rate of all European countries for the same year. aPM_2.5_ ratio (PMR) represents the ratio between a country's aPM_2.5_ concentration for a given year and a median aPM_2.5_ level of all European countries for the same year.

$$DARR_{1990} = \frac{{DALY rate_{1990} \left( {country} \right)}}{{median \left[ {DALY rate_{1990} \left( {Europe} \right)} \right]}}$$

$$DARR_{2019} = \frac{{DALY rate_{2019} \left( {country} \right)}}{{median \left[ {DALY rate_{2019} \left( {Europe} \right)} \right]}}$$

$$DARR change_{1990 - 2019} = \frac{{DARR_{1990} - DARR_{2019} }}{{DARR_{1990} }}$$

$$PMR_{1990} = \frac{{aPM_{2.5_{1990}} \left( {country} \right)}}{{median \left[ {aPM_{2.5_{1990}} \left( {Europe} \right)} \right]}}$$

$$PMR_{2019} = \frac{{aPM_{2.5_{2019}} \left( {country} \right)}}{{median \left[ {aPM_{2.5_{2019}} \left( {Europe} \right)} \right]}}$$

$$PMR change_{1990 - 2019} = \frac{{PMR_{1990} - PMR_{2019} }}{{PMR_{1990} }}$$

YLL rate ratio (YRR) represents the ratio between a country's YLL rate for a given year and the median YLL rate of all European countries for the same year.

Death rate ratio (DRR) represents the ratio between a country's death rate for a given year and the median death rate of all European countries for the same year.

### Statistical analyses

Burden of disease (e.g. attributable DALYs or mortality) calculation requires a few factors to be taken into account: spatial and temporal estimates of population-weighted exposure, TMREL, estimation of relative risk from exposure, as well as estimates of DALYs and deaths for diseases linked causally to air pollution. First, the data for relative risk and estimates of exposure of the population are combined which allows for the calculation of PAF, a proportion of DALYs and deaths in a population that can be attributed to exposure (e.g. to air pollution) above TMREL. Finally, the number of DALYs and deaths for certain diseases are multiplied by PAF and the end value gives an estimation of the burden attributable to the exposure. Specifically, we used DALYs, mortality and YLL attributable to air pollution overall, as well as to aPM_2.5_, household air pollution and ozone. A more detailed description of these methods can be found in GBD Study 2015 and 2019^6,22^.

Since the primary and final time points of our study were 1990 and 2019, we determined DARR and PMR for each European country for both years. We then defined change in DARR and PMR between those two time points as a new variable (DARR change and PMR change) and used it to quantify each country's ability to decrease its aPM_2.5_ and DALY values to at least the extent of the European aPM_2.5_ concentration and DALY median decrease between those two temporal points. Furthermore, 1990 and 2019 also respectively represent the largest and smallest value of European median value for aPM_2.5_ rate and DALY rate with other values following a linear decrease during that 29-year period, starting from 1990 (Supplementary Figure S1).

If DARR change or PMR change are positive, a country shows a reduction in its aPM_2.5_ or DALY values minimally to the extent of the European aPM_2.5_ concentration or DALY median decrease, but if DARR change or PMR change are negative, a country cannot follow the European median reduction. However, countries which improved their own DALY rate and aPM_2.5_ concentration may still be represented with a negative DARR change or PMR change if that improvement is lower than the extent of the European DALY or aPM_2.5_ concentration median decrease. Using this formula, we also calculated YRR and DRR, as well as their change between 1990 and 2019. Other variables in different time points can also be analysed in a similar manner. A visual representation of the calculation can be found in Supplementary Figure S6.

For each country, all variables are represented as a numerical value along with a 95% uncertainty interval (95% UI). For descriptive analysis of subgroups and all countries together, median and interquartile range (IQR) are predominantly used due to significant dataset variability and deviation from Gaussian distribution, unless stated otherwise. On the other hand, the death number is the only variable presented as a cumulative death number of all countries within a certain subgroup.

Due to significant dataset variability and deviation from Gaussian distribution, dependence between variables (YLL and aPM_2.5_ concentration, PMR and DARR) was established using Spearman correlation test. Furthermore, Kruskal–Wallis test by ranks with Dunn’s multiple comparisons test was used to determine statistical significance between subgroups of countries classified by their GNI and SDI.

Multiple linear regression was used to predict the outcome of YLL rate attributable to air pollution for 2019 using air pollution-related explanatory variables and to further establish relationship between air pollution parameters and health outcomes. Explanatory variables used in the model were aPM_2.5_ and household air pollution (HAP) for 2019. Both explanatory and response variables were log-transformed (ln-transformed). Ozone was excluded from the model because it failed to meet the assumption of linear relationship with the response variable. Even after multiple data transformations, it seemed that ozone did not have any significant relationship with the response variable. Furthermore, forcing the ozone into the model did not produce any significant improvement in proportion of explained variance. Alpha value for all statistical tests was set at 0.05. Data was analysed using GraphPad Prism 9 and Statistica 13.5.

## Results

### Overall change of air quality parameters and morbidity and mortality estimates attributable to air pollution in Europe from 1990 to 2019

**Figure 1:**
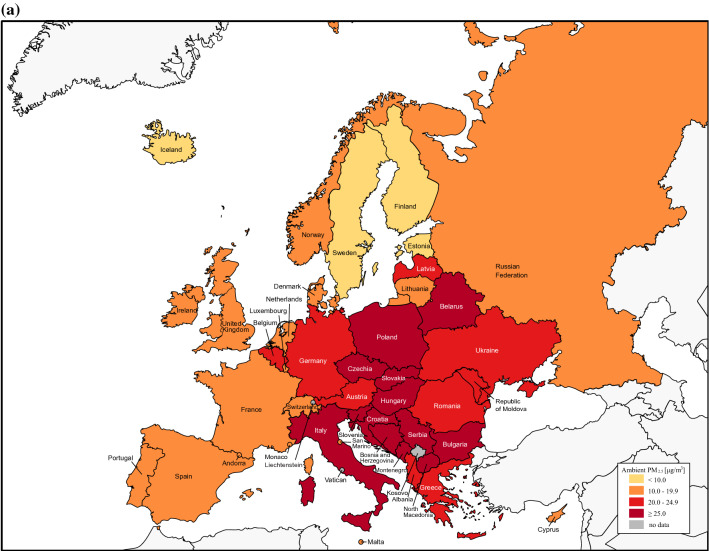

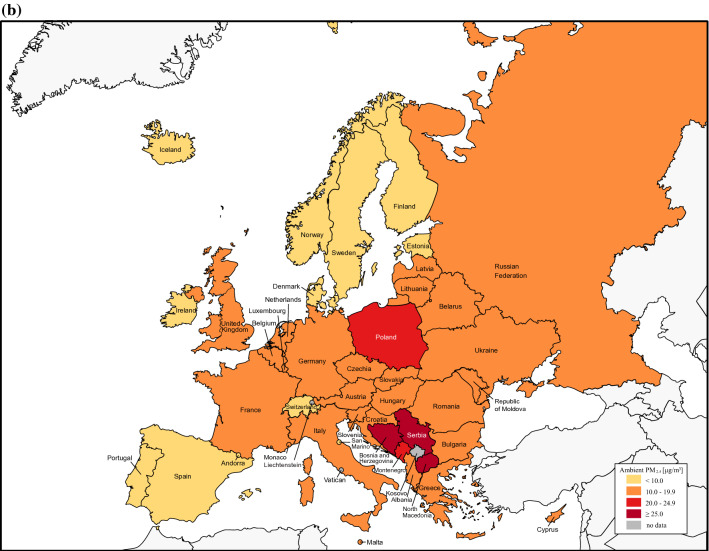

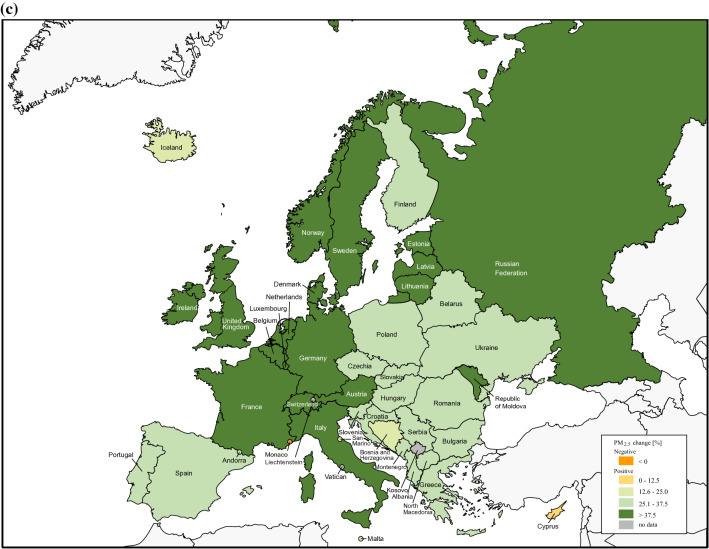
The average annual population-weighted aPM_2.5_ concentration in European countries for 1990 (**a**) and 2019 (**b**). Countries are categorised by parameters of the European Environment Agency for 2019 into groups based on aPM_2.5_ concentrations [μg/m^3^] in 2019: good (0–10.0), fair (10.0–19.9), moderate (20.0–24.9) and poor (≥ 25.0). Percent reduction in annual population-weighted aPM_2.5_ concentration in 2019 compared to 1990 (**c**). The figure was made in Adobe Illustrator (version 24.1., URL: https://www.adobe.com/products/illustrator.html).

The average aPM_2.5_ exposure of European countries was 20.8 μg/m^3^ (95% CI 18.3–23.2) in 1990, while in 2019 it was 33.7% lower at 13.8 μg/m^3^ (95% CI 12.0–15.6). All European countries reduced their annual population-weighted aPM_2.5_ concentration, except Monaco (Fig. 1, Supplementary Table S1). Western, Nordic and Baltic countries showed the biggest improvement in general, whereas progress was smaller for Eastern and Southeastern countries. On the other hand, average seasonal population-weighted ozone concentrations did not reduce as much as aPM_2.5_ with 44.7 ppb, a 6.5% decrease compared to 41.8 ppb in 2019 (Supplementary Figure S2).

**Figure 2:**
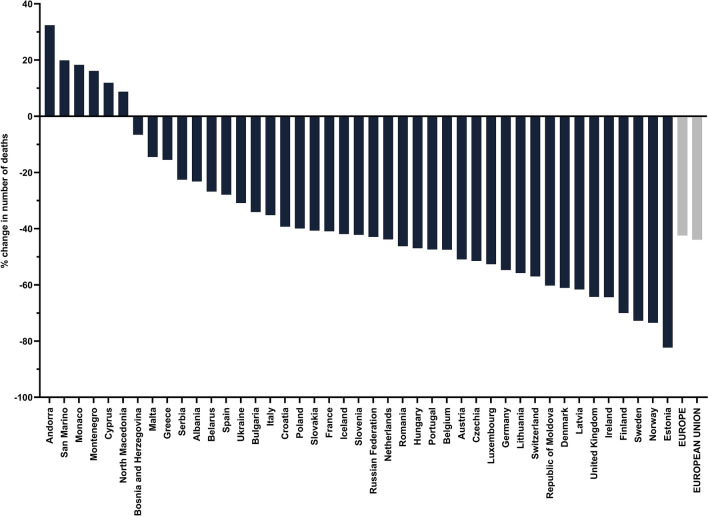
Percent change in number of deaths attributable to air pollution in 2019 compared to 1990.

**Figure 3:**
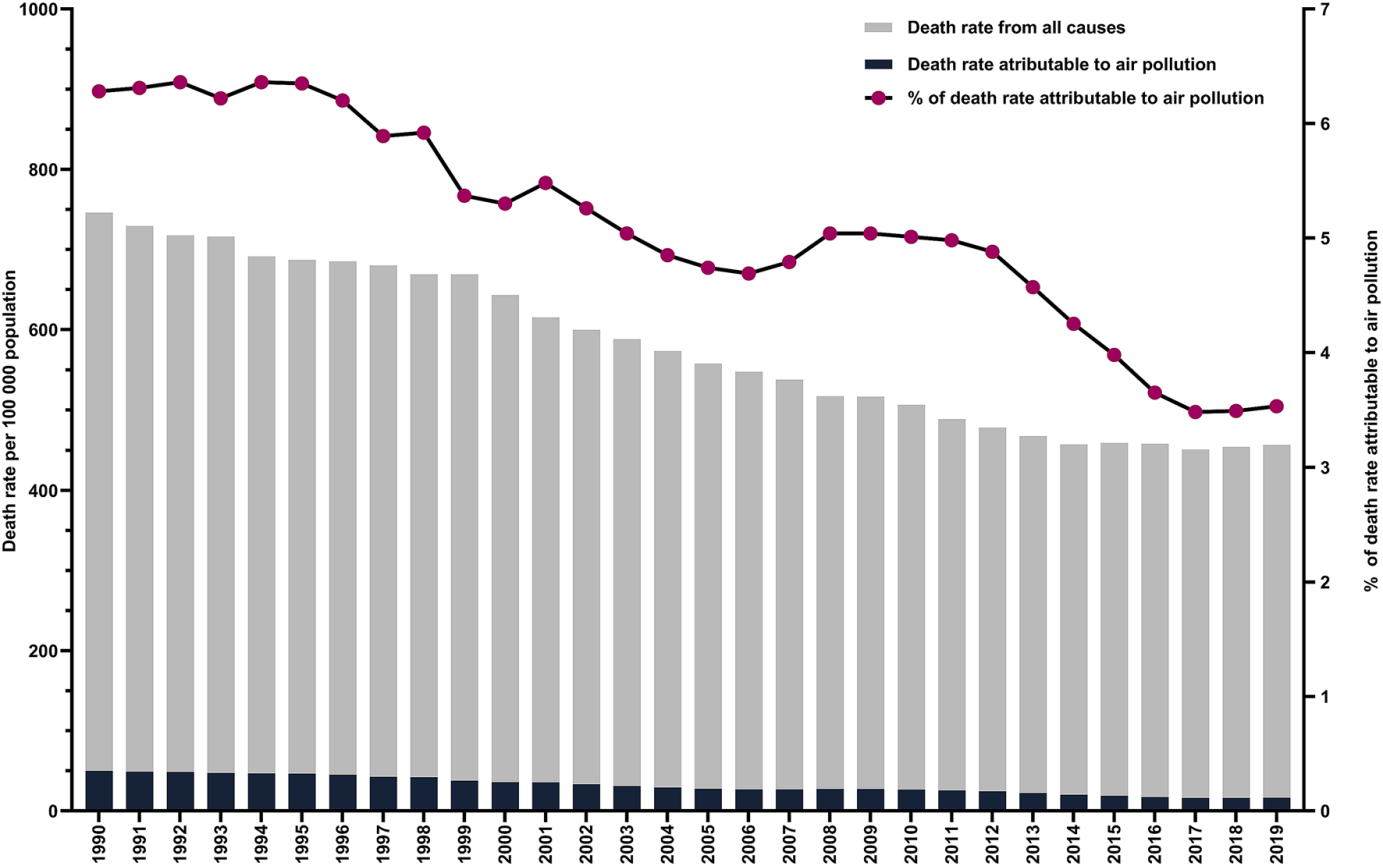
Median European death rate from 1990 to 2019.

We then analysed death number, death rates and DALY rates attributable to air pollution for 2019 in all 43 countries (Table 1). There were in total 368,006 deaths in Europe attributable to air pollution, a reduction of 271 046 (42.4%) compared to 1990 with 37 countries lowering their number of deaths (Fig. 2). In the same time period, an overall 43.9% decrease in total number of deaths attributable to air pollution was observed in the subset of EU countries. Estonia had the most significant decrease in mortality attributable to air pollution with a 82.3% reduction in 2019 when compared to 1990. It is followed by Norway and Sweden with 73.5% and 72.8% fewer deaths, respectively. During the 29-year time period, we also observed a decreasing trend of all-cause median death rate of all European countries, with a total reduction of 40.6% (Fig. 3). EU countries had a 1.3% lower death rate and 5.6% lower DALY rate attributable to air pollution (Supplementary Table S2) compared to all European countries. Furthermore, reduction of death rate attributable to air pollution was more pronounced when compared to overall death rate. It was 50.0 in 1990, whereas in 2019 it was 16.7, a 66.6% decrease.

**Table 1:** Deaths, age-standardised death rates, and DALYs rate attributable to air pollution in Europe in 2019.

Measure	Air pollution			Ambient particulate matter pollution			Household air pollution from solid fuels			Ambient ozone pollution		
	Deaths (thousands)	Median age standardised death rate per 100,000 (IQR)	Median age standardised DALY rate (IQR)	Deaths (thousands)	Median age standardised death rate per 100,000 (IQR)	Median age standardised DALY rate (IQR)	Deaths (thousands)	Median age standardised death rate per 100,000 (IQR)	Median age standardised DALY rate (IQR)	Deaths (thousands)	Median age standardised death rate per 100,000 (IQR)	Median age standardised DALY rate (IQR)
**Cause**												
All cause	368.0	16.7 (33.5)	403.4 (820.2)	332.7	39.7 (48.4)	382.2 (658.2)	19.5	0.2 (3.0)	3.8 (70.9)	17.3	1 (0.8)	15.4 (12.3)
Ischemic heart disease	164.3	6.5 (17.2)	133.5 (347.1)	155.1	6.0 (14.0)	124.8 (290.1)	9.2	0.1 (1.5)	1.0 (27.9)	N/A	N/A	N/A
Stroke	92.7	3.2 (8.7)	64.4 (202.8)	86.4	3.2 (7.8)	64.1 (187.1)	6.3	0.04 (0.8)	0.7 (17.9)	N/A	N/A	N/A
Tracheal, bronchus, and lung cancer	39.5	2.4 (2.1)	55.6 (56.4)	37.7	2.3 (1.9)	55.5 (44.5)	1.8	0.02 (0.2)	0.6 (5.2)	N/A	N/A	N/A
**Sex**												
Female	174.1	11.7 (24.9)	283.2 (570.9)	156.5	34.2 (44.2)	274.9 (467.2)	11.2	0.1 (2.6)	3.3 (57.6)	7.1	0.6 (0.6)	8.1 (8.5)
Male	193.9	23.6 (43.7)	537.7 (1107.6)	176.3	45.2 (51.2)	500.1 (892.3)	8.4	0.2 (3.4)	4.4 (86.6)	10.2	1.5 (1.1)	23.2 (16.8)
**Age**												
> 70 years	249.9	226.7 (308.1)	3034.8 (4523.9)	223.5	197.5 (284.5)	2731.6 (4079.0)	13.1	1.9 (31.3)	27.4 (455.3)	14.6	11.1 (11.4)	140.9 (128.8)
< 5 years	1	1.9 (1.8)	168.2 (163.1)	0.9	1.8 (1.7)	163.8 (151.7)	0.1	0.02 (0.3)	1.9 (23.0)	N/A	N/A	N/A

IQR, interquartile range. Certain data for ozone marked with "N/A" was not available in GDB 2019.

IHD was the primary cause of death in Europe making up 44.6% of all deaths attributable to air pollution. Stroke and TBL cancer had a smaller contribution to the total death number with 25.2% and 10.7%, respectively. When analysing the air pollution parameters, the majority (90.4%) of all deaths were associated with aPM_2.5_ pollution. Therefore, due to the high share in total number of deaths among all air pollutants, we primarily focused on analysing effects of aPM_2.5_ on health.

**Figure 4:**
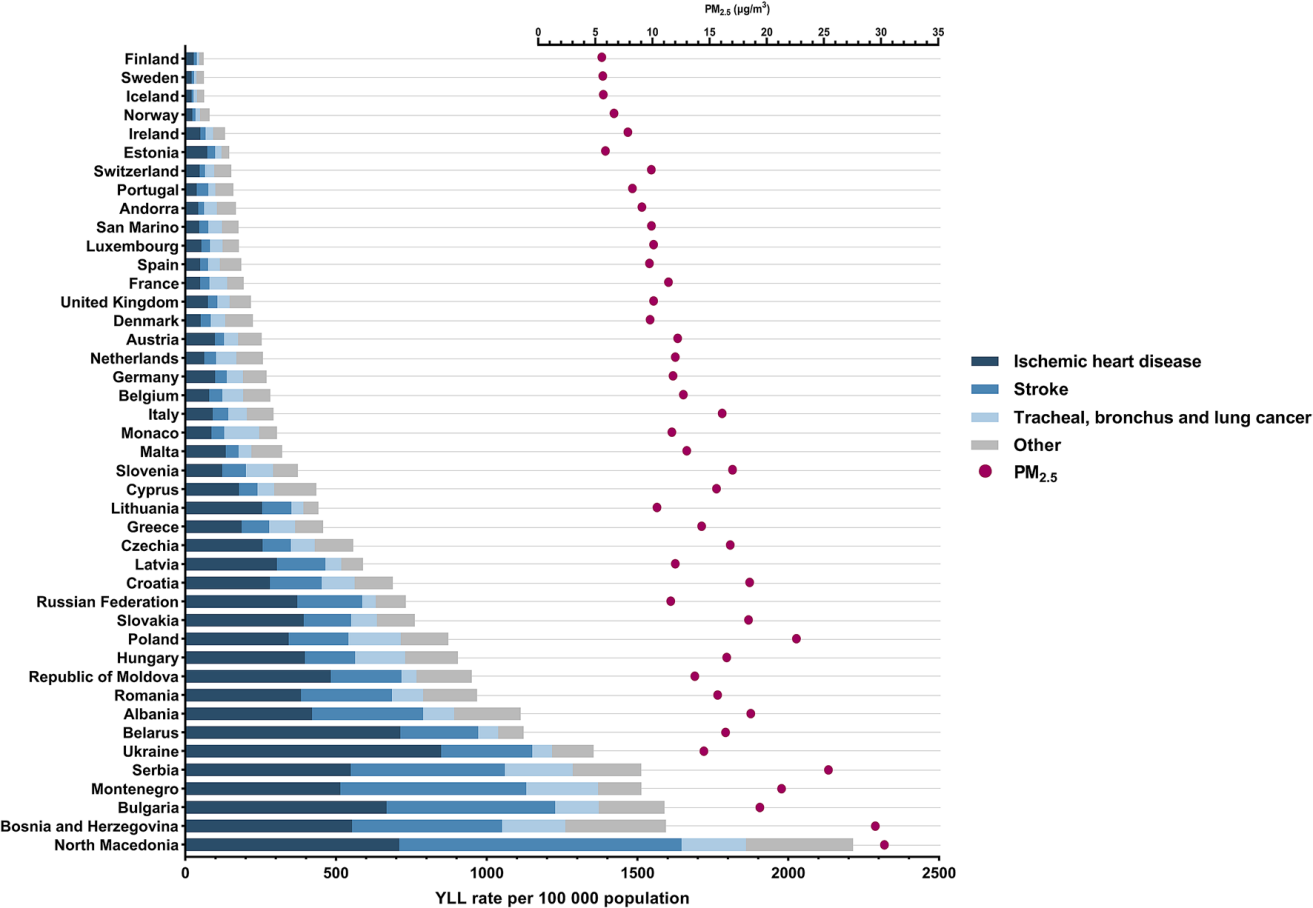
Age-standardised YLL rate per 100,000 and average annual population-weighted aPM_2.5_ in 2019.

Since we showed that aPM_2.5_ concentration in Europe decreased, we aimed to determine if the contribution of death rate attributable to air pollution in all-cause death rate also decreased. In 1990, it was 6.3% while in 2019 it was 3.5%, a reduction of 44.4%. We then aimed to explore how air pollution impacts premature mortality. Our analysis showed that a total of 24,917 years of life were lost per 100,000 population in Europe in 2019 due to health conditions associated with air pollution exposure (Fig. 4). This is a 63% decrease compared to 1990 (YLL = 67 258). Among all European countries in 2019, Finland had both the lowest YLL rate (60.9) and aPM_2.5_ concentration (5.6 μg/m^3^), while North Macedonia had the highest YLL rate (2214.9) and aPM_2.5_ concentration (30.3 μg/m^3^). Furthermore, a strong positive correlation was observed between aPM_2.5_ concentration and YLL rate (r = 0.911, *p* < 0.0001) (Supplementary Figure S3).

With IHD being the primary cause of death attributable to air pollution in Europe, we analysed YLL rate among European countries and observed that IHD also contributed the most with 41.2% of total YLL rate attributable to air pollution in 2019. IHD contributed the most to the YLL rate in Belarus (63.6%), while in Denmark it had the lowest contribution (22.9%).

### Progress of each country compared to Europe overall in terms of DALYs and aPM_2.5_

**Figure 5:**
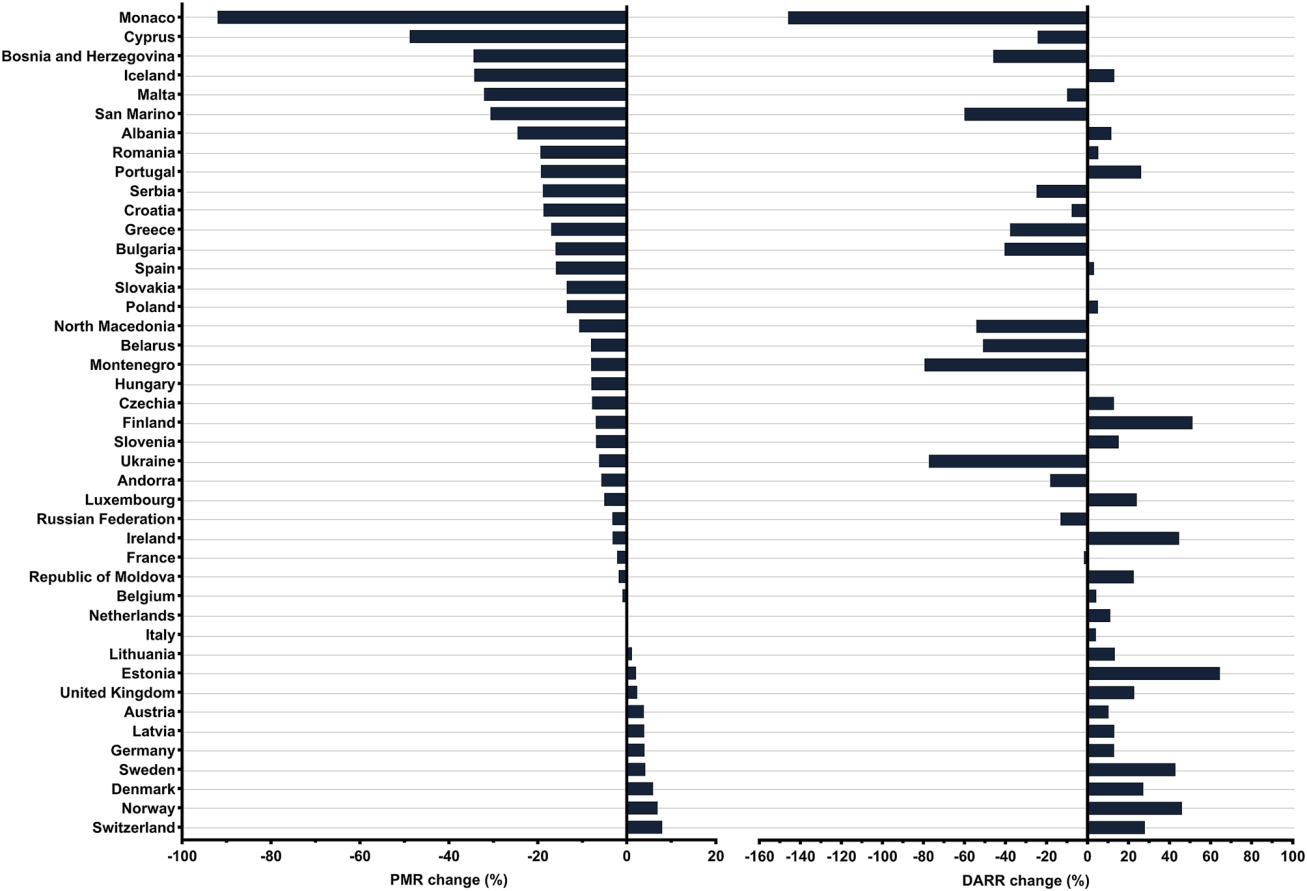
DARR change (%) and PMR change (%) for European countries.

To evaluate progress and the dynamics of reduction of air pollution parameters, we aimed to determine how efficient each European country was in reducing its DALY and aPM_2.5_ values to at least the extent of the European median decrease from 1990 to 2019. Therefore, European countries were compared using the DARR change attributable to air pollution in the first and final year of the analysed period. This value represents a difference between each country's DALY rate attributable to air pollution for the two given years (1990 and 2019) divided by the median European DALY for 1990. Figure 5 shows that 26 of 43 European countries improved their DARR, i.e. had a positive DARR change. These countries decreased their DALY rate attributable to air pollution minimally to the extent of the European median reduction. Estonia and Finland had the greatest improvement by decreasing DARR from 1990 to 2019 by 64.5% and 51.2%, respectively. On the other hand, 17 of 43 countries increased their DARR and are represented with a negative DARR change. Therefore, they have not been able to reduce their DALY values to at least the extent of the European median decrease. Monaco and Montenegro had the most negative values with an increase of 146.0% and 79.5%, respectively.

PMR was also determined for each European country. Similarly to DARR change, PMR change was used as a measure of each country's ability to decrease its aPM_2.5_ value minimally to the extent of the European median reduction. Although all countries (except Monaco) decreased their aPM_2.5_ level in 2019 compared to 1990, 31 of 43 European countries had a negative PMR change and an unfavorable trend of increasing PMR in 2019 compared to 1990, thus not reducing its aPM_2.5_ concentration to at least the extent of decrease in the European median. Monaco had the most prominent PMR change with an increase in PMR of 92% while other countries showed an increase of less than 50%. Although Finland increased its PMR by 7%, it still remained the country with the lowest aPM_2.5_ level among European countries in 2019 and second lowest in 1990. Italy and Netherlands had a neutral PMR change (0%) because they had equal PMRs both in 1990 and 2019. 10 of 43 countries had a positive PMR change and favorable trend of decreasing aPM_2.5_ values to at least the extent of the European median decrease with Switzerland having the largest reduction in PMR of 8%, followed by Norway and Denmark with a decrease of 6.9% and 5.9%, respectively. Furthermore, using the Spearman test, positive correlation between DARR and PMR was established both for 1990 (r = 0.854) and 2019 (r = 0.921). Using this calculation, YRR and DRR were also analysed (Supplementary Table S3).

In order to explore whether aPM_2.5_ and HAP can significantly predict YLL attributable to air pollution for 2019, a multiple regression was performed. Our model statistically significantly predicted YLL values and also explained a significant proportion of variance in YLL (R^2^ = 0.885, F(2,40) = 154.116, *p* < 0.0001). Both aPM_2.5_ (b = 1.623, *p* < 0.0001) and HAP (b = 0.150, *p* < 0.0001) were statistically significant predictors. The final model was:

$${\text{log}}\left( {{\text{YLL}}} \right) = {2}.{476} + {1}.{623} \times {\text{log}}\left( {{\text{aPM}}_{{{2}.{5}}} } \right) + 0.{15} \times {\text{log}}\left( {{\text{HAP}}} \right)$$

As a practical example of this model, it predicts that for a 10% increase in aPM_2.5_, YLL increases by 16.7%. Furthermore, for a 10% increase in household air pollution, YLL increases by 1.4%.

### Impact of economic and social factors on air pollution-related disease burden

**Figure 6:**
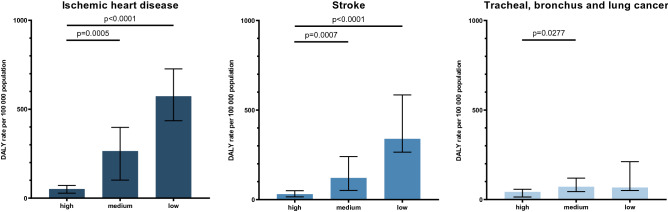
Comparison of countries by SDI and DALY rate attributable to air pollution for IHD, stroke and TBL cancer in 2019. Countries are categorised by SDI into three categories: high SDI, medium SDI and low SDI. Groups are represented as median with interquartile range. Statistical analysis by Kruskal–Wallis test by ranks with Dunn’s multiple comparisons test. Bonferroni correction for multiple tests was used to adjust significance values.

Then, we aimed to determine if there was a significant difference in aPM_2.5_ concentration and DALY rate attributable to air pollution based on a country's SDI. Thus, countries were grouped by their SDI in three categories (Fig. 6). Kruskal–Wallis test showed significant differences between groups for all three diseases: IHD (H(2) = 22.344, *p* < 0.0001), stroke (H(2) = 21.847, *p* < 0.0001) and TBL cancer (H(2) = 8.258, *p* = 0.016). When analysing differences between groups for IHD and stroke attributable to air pollution in 2019, low SDI countries showed more than a 11-fold higher DALY rate both for IHD and stroke when compared to high SDI countries. Furthermore, more than a fivefold higher DALY rate for IHD and nearly fourfold higher DALY rate for stroke was observed in medium SDI countries compared to high SDI countries. When looking at TBL cancer, DALY rate showed lower variability among groups, but a statistically significant difference (*p* = 0.028) in DALY rate was observed when comparing high SDI to medium SDI countries. Significant differences (H(2) = 16.844, *p* = 0.0002) were also observed between groups of countries when comparing their aPM_2.5_ concentrations (Supplementary Figure S4B).

In addition, we also compared groups of countries based on their GNI to assess if a country’s income might be a differentiating factor in terms of DALY rate attributable to air pollution. There were significant differences between groups for all three diseases: IHD (H(3) = 28.038, *p* < 0.0001), stroke (H(3) = 28.963, *p* < 0.0001) and TBL cancer (H(3) = 15.550, *p* = 0.001). We showed that groups of countries with lower GNI had significantly higher DALY compared to groups of countries with higher GNI (Supplementary Figure S4A). This effect is most prominent when looking at countries in the lowest economic bracket which have more than a 11-fold higher DALY rate for IHD and nearly 25-fold higher DALY rate for stroke when compared to very high income (VHI) countries. Furthermore, significant differences (H(3) = 19.918, *p* = 0.0002) were also observed between GNI groups when comparing their aPM_2.5_ concentrations (Supplementary Figure S4B). These findings align well with SDI comparisons, aided by a strong correlation between GNI and SDI (Spearman r = 0.893, *p* < 0.0001). Taken together, these comparisons show that countries with higher GNI and SDI have lower DALY rates attributable to air pollution compared to countries in lower GNI and SDI brackets.

## Discussion

Air pollution is the most important environmental risk to human health^27^. It is also perceived among Europeans as the second biggest environmental concern^28^. A growing interest in the topic of air pollution has led to public and political actions ultimately successfully reducing air pollution levels.

We showed that all European countries excluding Monaco decreased the concentration of the leading air pollutant aPM_2.5_ by up to 44% in 2019 compared to 1990. One of the reasons that Monaco is an exception to this positive trend might be the very high population density (more than 170-fold more than the average population density of the European Union) which influences the aPM_2.5_ value since the aPM_2.5_ exposure calculation accounts for population density. The overall decrease in Europe may partly be due to strong and proactive political legislations which have proved effective in curbing air pollution in many studies^29–31^. The Convention on Long-range Transboundary Air Pollution has successfully reduced air pollution in Europe, whereas the European Green Deal focuses on making Europe climate neutral by 2050^32^. The act empowers to prioritize a sustainable industry, energy efficiency by using clean energy, the importance of recycling, optimizing agriculture, and sustainable mobility^33^. Reductions in air pollution were closely followed by reduced DALYs and mortality attributable to air pollution improving the overall population health (Supplementary Figure S5). However, despite the progress made, air pollution remains an important pan-European public health issue with nearly ¾ of European countries still exceeding the annual WHO AQG for aPM_2.5_ pollution of 10 μg/m^3^ in 2019. This is especially important in the context of the ever-growing European population where urbanization is expected to increase from 74.7% to approximately 83.7% in 2050^19,34^.

Study from Boldo et al. with 23 European cities showed that life expectancy at age 30 would increase by a range between one month and more than two years if long-term exposure to aPM_2.5_ level was reduced to 15 μg/m^3^^35^. Also, a recent study from almost 1000 European cities calculated that 51,213 deaths per year could be avoided if PM_2.5_ exposure was compliant with WHO air pollution guidelines^36^.

As a result of improved air quality, more than 85% of countries in Europe had a lower number of deaths attributable to air pollution in 2019 compared to 1990. Also, the overall number of deaths attributable to air pollution was lowered by more than 270,000 (42.4%). Interestingly, the share of death rate attributable to air pollution in the overall all-cause death rate in Europe decreased more than the overall death rate, thus outpacing it from an approximate 6% share in 1990 to nearly 3.5% in 2019. On the other hand, despite reducing air pollution, Southeast European countries Bosnia and Herzegovina and North Macedonia still had the highest aPM_2.5_ levels in Europe in 2019, three times higher than the WHO AQG. Our findings align with Lelieveld et al. who also found that air pollution-related mortality per capita was high in Eastern European countries, especially concerning cardiovascular mortality^37^. Death rates in Bosnia and Herzegovina and North Macedonia were five-fold and seven-fold higher than the European median, respectively. Their DALY rates were also the highest in Europe, multiple times higher than both the EU and European median and up to 32 times higher than Iceland, which had the lowest DALY rate, thus indicating room for improvement. In another study, Lehtomäki et al. showed that Iceland had the lowest death rate among five Nordic countries, all of which have relatively low levels of air pollution, and generally meet the EU guideline values^38^. Furthermore, for a deeper understanding of time trends in burden estimates, a decomposition of total changes in DALY rates or mortality over time for each European country could be performed, taking into account the contribution of population size change, age, cause specific-mortality rates (excluding the effect of air pollution), and air pollution exposure. These analyses could provide a useful overview of how each factor contributes to changes in DALY rates or mortality, and thus inform policy makers, as well as health officials, about potentially implementing specific measures to address the factors contributing most to disease burden. Decomposition methods have been previously reported in GBD studies, as well as papers that focused primarily on this method, which due to its significant comprehensiveness, was not within the scope of our research^6,39,40^.

As a summary measure of premature mortality, YLL has been highly associated with air pollution and each year more than 200 million years of life are lost due to air pollution globally^41^. Our results align closely since we showed a strong positive association between aPM_2.5_ concentration and YLL. One study using data from 72 Chinese cities estimated that for every 10 μg/m^3^ increase in PM_2.5_ an additional 0.43 years of life are lost, whereas PM_2.5_ levels in accordance with WHO AQG would result in 0.14 years of gain in life expectancy^42^. To get a better and more comprehensive understanding of the relationship between disease burden metrics such as YLL and aPM_2.5_ and household air pollution, the model we created came very useful. Due to its significant prediction of YLL attributable to air pollution using aPM_2.5_ and household air pollution, we think this might be of good use to policymakers and researchers in the field to reliably predict YLL based on trends of air pollution components, especially during a period of years. Furthermore, since the model predicts that a 10% increase in aPM_2.5_ would result in YLL increase of 16.7%, whereas a 10% increase in household air pollution would increase YLL by only 1.4%, it strongly supports that aPM_2.5_ has the predominant impact on disease burden, thus pointing to the importance of curbing this air pollution parameter.

Comparing the results to the overall trend in the region is also a significant factor regarding not only a country’s environmental consciousness, but also the ability to leverage resources to combat air pollution. Both PMR change and DARR change are important to see if the country is reducing its aPM_2.5_ concentration and DALY values at least at the pace of the European region between 1990 and 2019. Despite almost all countries decreasing their aPM_2.5_ concentrations in 2019**,** only 10 countries actually did that to at least the extent of the European median decrease. Sweden and Norway were among the countries with the lowest aPM_2.5_ concentrations in 1990, yet still managed to decrease their aPM_2.5_ values in 2019 more than the European median, thus having a positive PMR change. On the other hand, Bosnia and Herzegovina and Albania had among the most negative PMR changes in spite of reducing their aPM_2.5_ concentrations. Belis et al. identified energy production in inefficient coal-fueled power plants as one of the main sources of PM_2.5_ in the Western Balkans. Also, agriculture and residential combustion significantly affected PM_2.5_ levels^43^. Since some Western Balkan countries had among the highest aPM_2.5_ levels both in 1990 and 2019, more powerful ways of curbing air pollution are needed.

Similarly, we used DARR change to evaluate each country's progress in terms of reduction in DALY rates attributable to air pollution compared to the European median change. All 43 countries reduced their own DALY rate in 2019 compared to 1990, but less than 2/3 had a decrease to at least the extent of the European median reduction, thus having a positive DARR change. EU countries Finland, Sweden, and Estonia all had DARR change above 40%, while Monaco, Ukraine and Montenegro had the most negative DARR change, up to -146%. The wide disparity in terms of DARR change may be due to more strict environmental policies in EU countries, ambitious targets for emission reductions, and economic power and development^44^. Therefore, both aPM_2.5_ concentration and DALY rate, as well as DARR change and PMR change are complementary methods which should be used when evaluating changes in aPM_2.5_ concentration and DALY rate between countries. It is important to note that multiple diverse variables and geographical regions could used in this calculation, which gives it a breath of flexibility and applicability in various scenarios.

Taking into account social and economic factors using SDI and GNI in regard to air pollution-related health burden is an important metric. We showed that countries in the lowest SDI category in Europe had a higher aPM_2.5_ concentration compared to those in the highest. This aligns well with another study showing that the country's lower development status might be associated with overall poorer air quality^8^. Developing countries undergoing the process of intense urbanization and industrialization became the countries with the largest air pollution-related burdens in recent years. Furthermore, indoor air pollution originating from coal and biomass in the form of wood, dung and crop residues for domestic energy represents a major environmental and public health challenge in developing economies, especially in rural areas^45^. Even though we expected more developed countries to have lower air pollution parameters, we were surprised by how large some of the differences were when comparing countries. Low SDI countries had more than a 11-fold higher DALY rate both for IHD and stroke when compared to high SDI countries. This difference has even greater meaning when taking into account that stroke and IHD are two of the leading causes of death in the world. Similar striking differences were found when comparing countries by GNI, thus excluding educational attainment and total fertility rate which are part of SDI. Upper middle income (UMI) countries had more than a 11-fold higher DALY rate for IHD and nearly 25-fold higher DALY rate for stroke when compared to VHI countries. This big difference emphasizes a larger than expected gap between more and less developed countries in Europe and shows how disparities in controlling air pollution can negatively affect population health. This is a strong call to action, especially in lower developed countries, to double down on curbing air pollution considering that stroke, IHD and TBL cancer made up more than 80% of all European deaths attributable to air pollution in 2019 alone. The difference in economic power and DALYs might also be due to other factors such as a more comprehensive and stronger healthcare system in wealthier countries. A study showed that better and more extensive quality of healthcare is needed to improve patient outcomes, especially since 63.8% of deaths in Eastern Europe occurred due to use of poor-quality services^46^. The somewhat smaller statistical significance for TBL cancer might be due to cancer being a complex heterogeneous disease which can be attributable to both genetics and lifestyle and thus various factors might be triggering its genesis in different and unequal manners^47^.

With cooperation at intra- and inter-national levels, strong policies could be implemented at curbing both air pollution and premature mortality and morbidity, while serving as a catalyst for economic development and promotion of healthy lifestyle^48^. Emissions from vehicles could be reduced by prioritizing green and sustainable forms of transport such as rapid and optimized urban and international transport, cycling, as well as implementing stricter vehicle emissions standards and working on more efficient engine technologies^48,49^. Making cities more compact and with energy efficient homes, optimizing urban transport and waste management will be of utmost importance to mitigate air pollution increase. Improving the management of agricultural waste and livestock manure, while reducing agriculture field burning and promoting healthy diets low in processed meat and rich in plant-based food will keep the food production environmentally sustainable^50^. Despite improvements in wastewater management and pollution abatement technology, industry is still a significant source of pollutant releases in Europe^51^. Further implementation of clean technologies, filters, and recovery of gas released during fossil fuel production are recommended to optimize soil, water and air quality management^52^. Public actions and national robust policies could have a long-lasting impact on bringing down air pollution, as well as growing human health and welfare.

Our study has several limitations. First, important causes of death like chronic obstructive pulmonary disease, dementias, diabetes and kidney disease were not individually addressed in the context of air pollution-related morbidity and mortality. Second, DARR change and PMR change might not give a clear indication of the country's progress in certain conditions. A negative DARR change and/or PMR change might underestimate the country's progress since it could already have low DALY rates or aPM_2.5_ values. Third, estimates on levels of aPM_2.5_ and ozone might be skewed given the smaller numbers and less well diversified locations of air quality monitoring stations, as well as the spread of air pollution from other countries through changes in wind pattern with temperature^53,54^. Also, GBD study estimates of household air pollution include solid fuels used for cooking, but not for heating. Fourth, availability of primary data is a major limitation of the GBD study and as such applies here for mortality and morbidity estimates, along with other general limitations described in the GBD Study 2019^22^. Fifth, decomposing the total changes in mortality or DALY rates over time for each European country, taking into account the contribution of population size change, age, cause specific-mortality rates (excluding the effect of air pollution), and air pollution exposure was not performed which could have given the paper a deeper understanding of the factors influencing them the most. Sixth, recommendations on how to reduce air pollution might not be feasible for every country in the same way due to different dominant industries and economic power. Finally, air pollution, as well as mortality and morbidity estimates for each country might not be representative for all the country's regions. Also, specific air pollution-related medical conditions might not be represented in an equal manner in the whole population and certain subpopulations might be more or less affected. Even with these limitations, our study provides a useful overview and analysis regarding the health effects of air pollution in Europe using the most recent data available.

In conclusion, Europe made significant progress in decreasing aPM_2.5_ concentration, mortality and disease burden attributable to air pollution in the last three decades. However, nearly 75% of Europeans still live in areas where aPM_2.5_ concentration do not meet WHO AQG. Even though implementing air pollution reduction measures may be a significant challenge for some countries, with population growth and increased urbanization in Europe, air quality should be prioritized for long term economic growth and improved overall population health.

## Supplementary Information

Supplementary Information.

## Data Availability

The dataset regarding overall, as well as ischemic heart disease (IHD), stroke, and tracheal, bronchus and lung cancer-specific disability adjusted life years (DALYs), years of life lost (YLL) and mortality attributable to air pollution for 43 European countries between 1990 and 2019 are available in the Global Burden of Disease 2019 repository which can be found on this web page: http://www.healthdata.org/gbd/2019. Socio-demographic index for each country can be found in the same database. The dataset regarding concentrations of ambient particulate matter less than 2.5 microns in size, ozone, and household air pollution from solid fuels were obtained from State of Global Air 2020 which can be found on this web page: https://www.stateofglobalair.org/. The dataset regarding the gross national income of each European country was obtained from the World Bank Classification and can be found on this web page: https://data.worldbank.org/indicator/NY.GNP.PCAP.CD. Other data used in this manuscript can be found within the reference section.

## References

[1] Ambient (outdoor) air pollution. World Health Organization. https://www.who.int/news-room/fact-sheets/detail/ambient-(outdoor)-air-quality-and-health (2018).

[2] Rafaj P (2018). Outlook for clean air in the context of sustainable development goals. Glob. Environ. Change.

[3] The top 10 causes of death. World Health Organization. https://www.who.int/news-room/fact-sheets/detail/the-top-10-causes-of-death (2020).

[4] Global Burden of Disease Collaborative Network. Global Burden of Disease Study 2019 (GBD 2019) Results. Institute for Health Metrics and Evaluation. http://ghdx.healthdata.org/gbd-results-tool (2020).

[5] Air Pollution. World Health Organization. https://www.who.int/health-topics/air-pollution#tab=tab_1 (2020).

[6] Cohen AJ (2017). Estimates and 25-year trends of the global burden of disease attributable to ambient air pollution: an analysis of data from the Global Burden of Diseases Study 2015. Lancet.

[7] World Bank; Institute for Health Metrics and Evaluation. The cost of air pollution: Strengthening the economic case for action. World Bank. https://openknowledge.worldbank.org/handle/10986/25013 (2016).

[8] Mannucci PM, Franchini M (2017). Health effects of ambient air pollution in developing countries. Int. J. Environ. Res. Public Health.

[9] Air pollution. European Environment Agency. https://www.eea.europa.eu/themes/air/intro (2020)

[10] European Environment Agency. Air quality in Europe: 2016 Report. European Environment Agency. https://www.eea.europa.eu/publications/air-quality-in-europe-2016 (2016).

[11] Commission Staff Working Document — Impact Assessment. European Commission. http://ec.europa.eu/environment/archives/air/pdf/Impact_assessment_en.pdf (2013).

[12] Air Quality in Europe: 2018 Report. European Environment Agency. https://www.eea.europa.eu/publications/air-quality-in-europe-2018 (2018).

[13] Anenberg SC, Achakulwisut P, Brauer M, Moran D, Apte JS, Henze DK (2019). Particulate matter-attributable mortality and relationships with carbon dioxide in 250 urban areas worldwide. Sci. Rep..

[14] The European environment — state and outlook 2020: Knowledge for transition to a sustainable Europe. European Environment Agency. https://www.eea.europa.eu/publications/soer-2020 (2019).

[15] Xu X (2011). Long-term exposure to ambient fine particulate pollution induces insulin resistance and mitochondrial alteration in adipose tissue. Toxicol. Sci..

[16] Xing YF, Xu YH, Shi MH, Lian YX (2016). The impact of PM2.5 on the human respiratory system. J. Thorac. Dis..

[17] Correia AW, Pope CA, Dockery DW, Wang Y, Ezzati M, Dominici F (2013). Effect of air pollution control on life expectancy in the United States: An analysis of 545 U.S. counties for the period from 2000 to 2007. Epidemiology.

[18] Kloog I, Ridgway B, Koutrakis P, Coull BA, Schwartz JD (2013). Long- and short-term exposure to PM2.5 and mortality: Using novel exposure models. Epidemiology.

[19] Urban population (% of total population) — European Union. World Bank. https://data.worldbank.org/indicator/SP.URB.TOTL.IN.ZS?locations=EU (2018).

[20] Jerrett M (2009). Long-term ozone exposure and mortality. N. Engl. J. Med..

[21] Bekkar B, Pacheco S, Basu R, DeNicola N (2020). Association of air pollution and heat exposure with preterm birth, low birth weight, and stillbirth in the US: A systematic review. JAMA Netw. Open..

[22] GBD 2019 Diseases and Injuries Collaborators. Global burden of 369 diseases and injuries in 204 countries and territories, 1990–2019: A systematic analysis for the Global Burden of Disease Study 2019. *Lancet*. **396,** 1204–1222 (2020). 10.1016/S0140-6736(20)30925-9 PMC7567026 33069326

[23] Health Effects Institute. State of Global Air 2020. Health Effects Institute. www.stateofglobalair.org (2020).

[24] World Development Indicators (Series: GNI per capita, Atlas method (current US$). The World Bank. http://data.worldbank.org/indicator (2020).

[25] Global Burden of Disease Cancer Collaboration (2017). Global, regional, and national cancer incidence, mortality, years of life lost, years lived with disability, and disability-adjusted life-years for 32 cancer groups, 1990 to 2015: A systematic analysis for the global burden of disease study. JAMA Oncol..

[26] The World Bank Atlas method — detailed methodology. World Bank. https://datahelpdesk.worldbank.org/knowledgebase/articles/378832-what-is-the-world-bank-atlas-method (2020).

[27] Chau TT, Wang KY (2020). An association between air pollution and daily most frequently visits of eighteen outpatient diseases in an industrial city. Sci. Rep..

[28] Special Eurobarometer 468: Attitudes of European citizens towards the environment. *EU Open Data Portal*. https://data.europa.eu/euodp/en/data/dataset/S2156_88_1_468_ENG (2021).

[29] Turnock ST (2016). The impact of European legislative and technology measures to reduce air pollutants on air quality, human health and climate. Environ. Res. Lett..

[30] Guerreiro CBB, Foltescu V, de Leeuw F (2014). Air quality status and trends in Europe. Atmos. Environ..

[31] Kuklinska K, Wolska L, Namiesnik J (2015). Air quality policy in the U.S. and the EU—A review. Atmos. Pollut. Res..

[32] Byrne A (2015). The 1979 convention on long-range transboundary air pollution: Assessing its effectiveness as a multilateral environmental regime after 35 years. Trans. Environ. Law.

[33] Communication from the Commission to the European Parliament, the European Council, The Council, the European Economic and Social Committee and the Committee of the Regions: The European Green Deal. European Commission. https://eur-lex.europa.eu/legal-content/EN/TXT/?uri=COM%3A2019%3A640%3AFIN (2019).

[34] Developments and Forecasts on Continuing Urbanisation. European Commission. https://knowledge4policy.ec.europa.eu/foresight/topic/continuing-urbanisation/developments-and-forecasts-on-continuing-urbanisation_en (2021).

[35] Boldo E (2006). Apheis: Health impact assessment of long-term exposure to PM 2.5 in 23 European cities. Eur. J. Epidemiol..

[36] Khomenko S (2021). Premature mortality due to air pollution in European cities: A health impact assessment. Lancet Planet. Health.

[37] Lelieveld J, Klingmüller K, Pozzer A (2019). Cardiovascular disease burden from ambient air pollution in Europe reassessed using novel hazard ratio functions. Eur. Heart J..

[38] Lehtomäki H, Geels C, Brandt J (2020). Deaths attributable to air pollution in nordic countries: Disparities in the estimates. Atmosphere.

[39] Cheng X (2020). Population ageing and mortality during 1990–2017: A global decomposition analysis. PLoS Med..

[40] Ščasný M, Ang BW, Rečkaa L (2021). Decomposition analysis of air pollutants during the transition and post-transition periods in the Czech Republic. Renew. Sustain. Energy Rev..

[41] Lelieveld J, Pozzer A, Pöschl U, Fnais M, Haines A, Münzel T (2020). Loss of life expectancy from air pollution compared to other risk factors: A worldwide perspective. Cardiovasc. Res..

[42] Qi J (2020). Potential gains in life expectancy by attaining daily ambient fine particulate matter pollution standards in mainland China: A modeling study based on nationwide data. PLoS Med..

[43] Belis CA (2019). Urban pollution in the Danube and Western Balkans regions: The impact of major PM2.5 sources. Environ. Int..

[44] Koolen CD, Rothenberg G (2019). Air pollution in Europe. Chemsuschem.

[45] Bruce N, Perez-Padilla R, Albalak R (2000). Indoor air pollution in developing countries: A major environmental and public health challenge. World Health Organ..

[46] Kruk ME, Gage AD, Joseph NT, Danaei G, García-Saisó S, Salomon JA (2018). Mortality due to low-quality health systems in the universal health coverage era: A systematic analysis of amenable deaths in 137 countries. Lancet.

[47] Chen Z, Fillmore CM, Hammerman PS, Kim CF, Wong K-K (2014). Non-small-cell lung cancers: A heterogeneous set of diseases. Nat. Rev. Cancer.

[48] Scovronick N (2015). Reducing Global Health Risks Through Mitigation of Short-Lived Climate Pollutants.

[49] Overview of Air Pollution from Transportation. United States Environmental Protection Agency. https://www.epa.gov/transportation-air-pollution-and-climate-change/learn-about-air-pollution-transportation (2021).

[50] Interventions: Agriculture. World Health Organization. https://www.who.int/airpollution/ambient/interventions/agriculture/en/ (2021).

[51] Air Quality in Europe: 2020 Report. European Environment Agency. https://www.eea.europa.eu//publications/air-quality-in-europe-2020-report (2020).

[52] Industrial pollution in Europe. European Environment Agency*.* https://www.eea.europa.eu/data-and-maps/indicators/industrial-pollution-in-europe-3/assessment (2021).

[53] De Sario M, Katsouyanni K, Michelozzi P (2013). Climate change, extreme weather events, air pollution and respiratory health in Europe. Eur. Respir. J..

[54] Kracht, O. *et al.* Spatial representativeness of air quality monitoring sites — Outcomes of the FAIRMODE/AQUILA intercomparison exercise. European Commission - Joint Research Centre. https://publications.jrc.ec.europa.eu/repository/handle/JRC108791 (2017).

