# Supplementary material for: Health impacts of air pollution exposure from 1990 to 2019 in 43 European countries
Source: Sci Rep. 2021 Nov 18;11:22516. doi: 10.1038/s41598-021-01802-5 (PMC8602675; doi:10.1038/s41598-021-01802-5)
Supplement: Supplementary file 1 — Supplementary Information. [file 41598_2021_1802_MOESM1_ESM.pdf]

Supplementary Figure S1

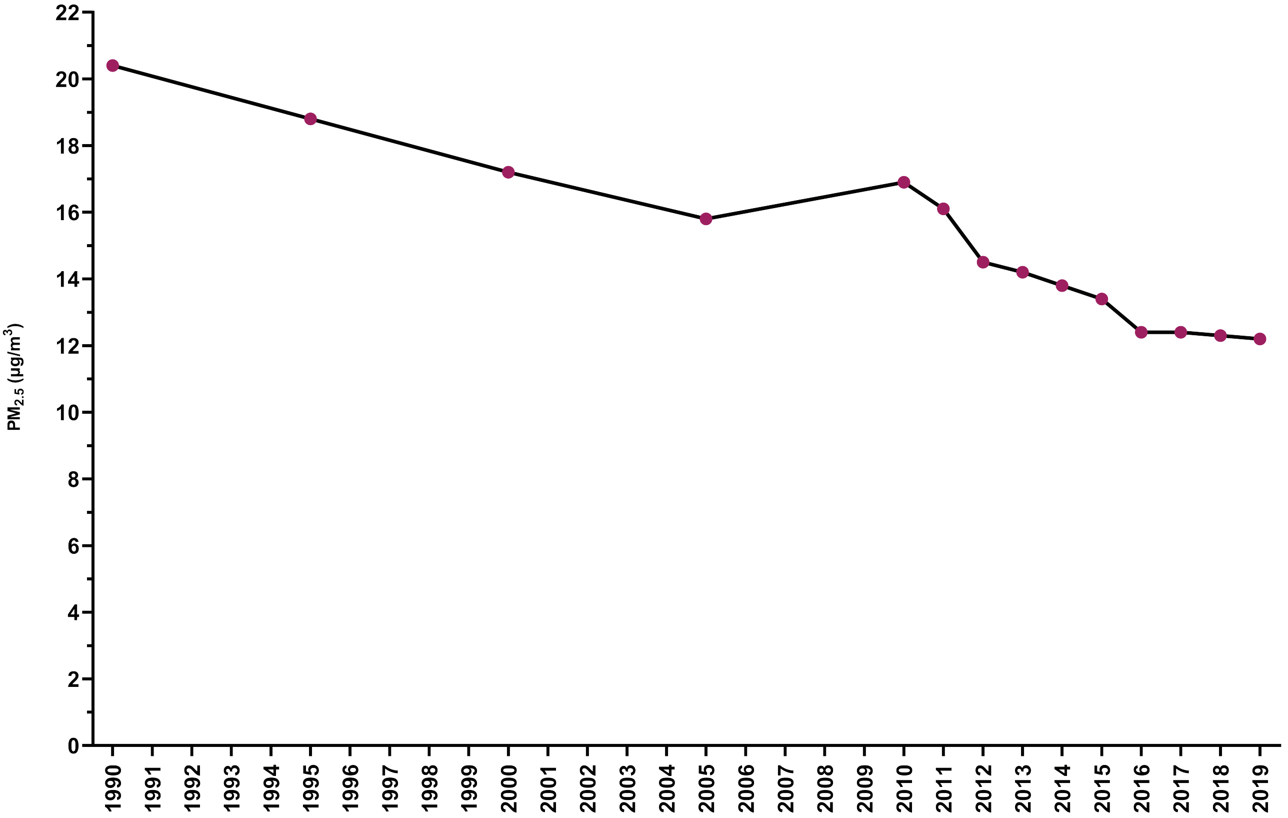

Supplementary Figure S2 (A)

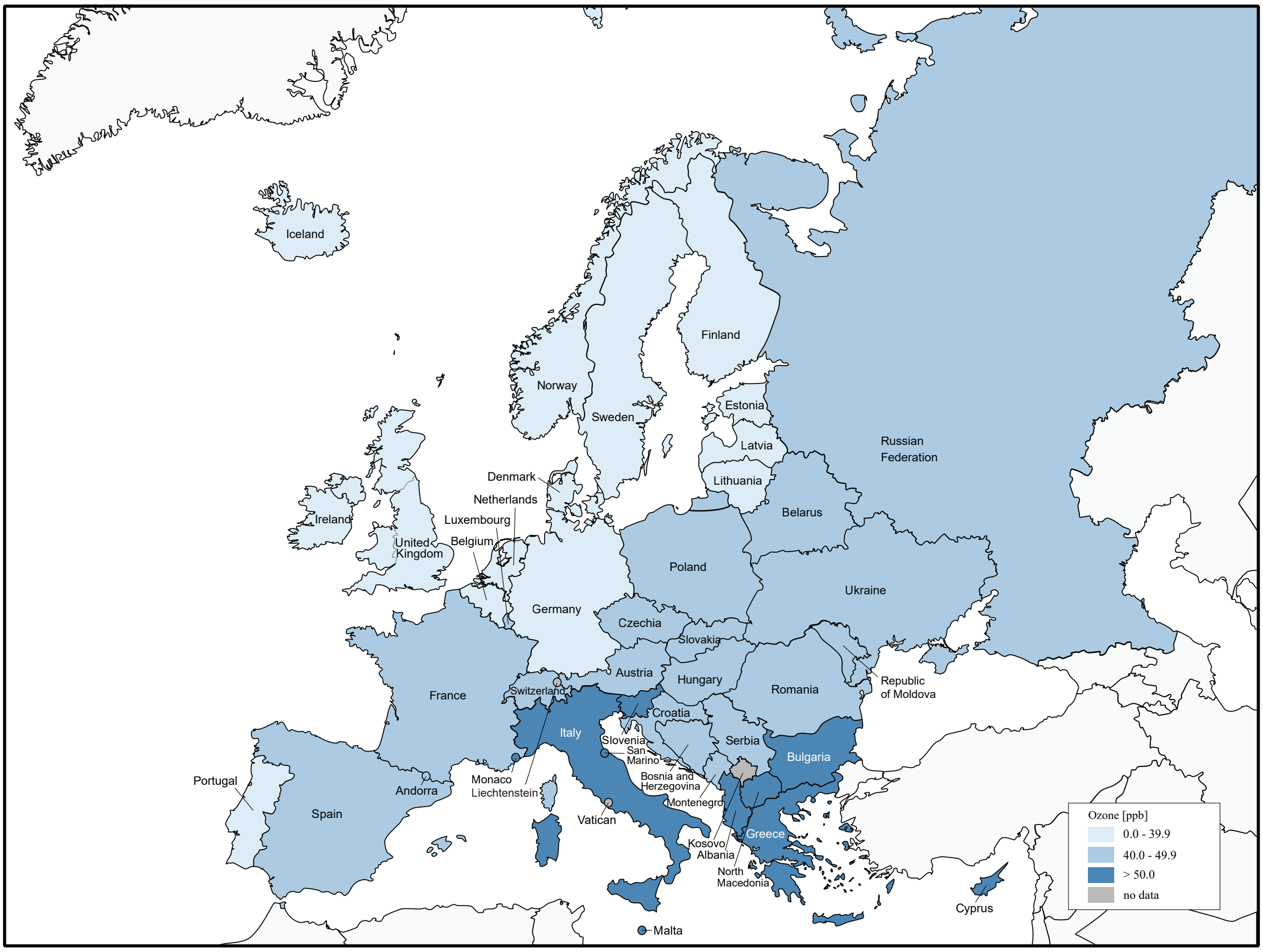

Supplementary Figure S2 (B)

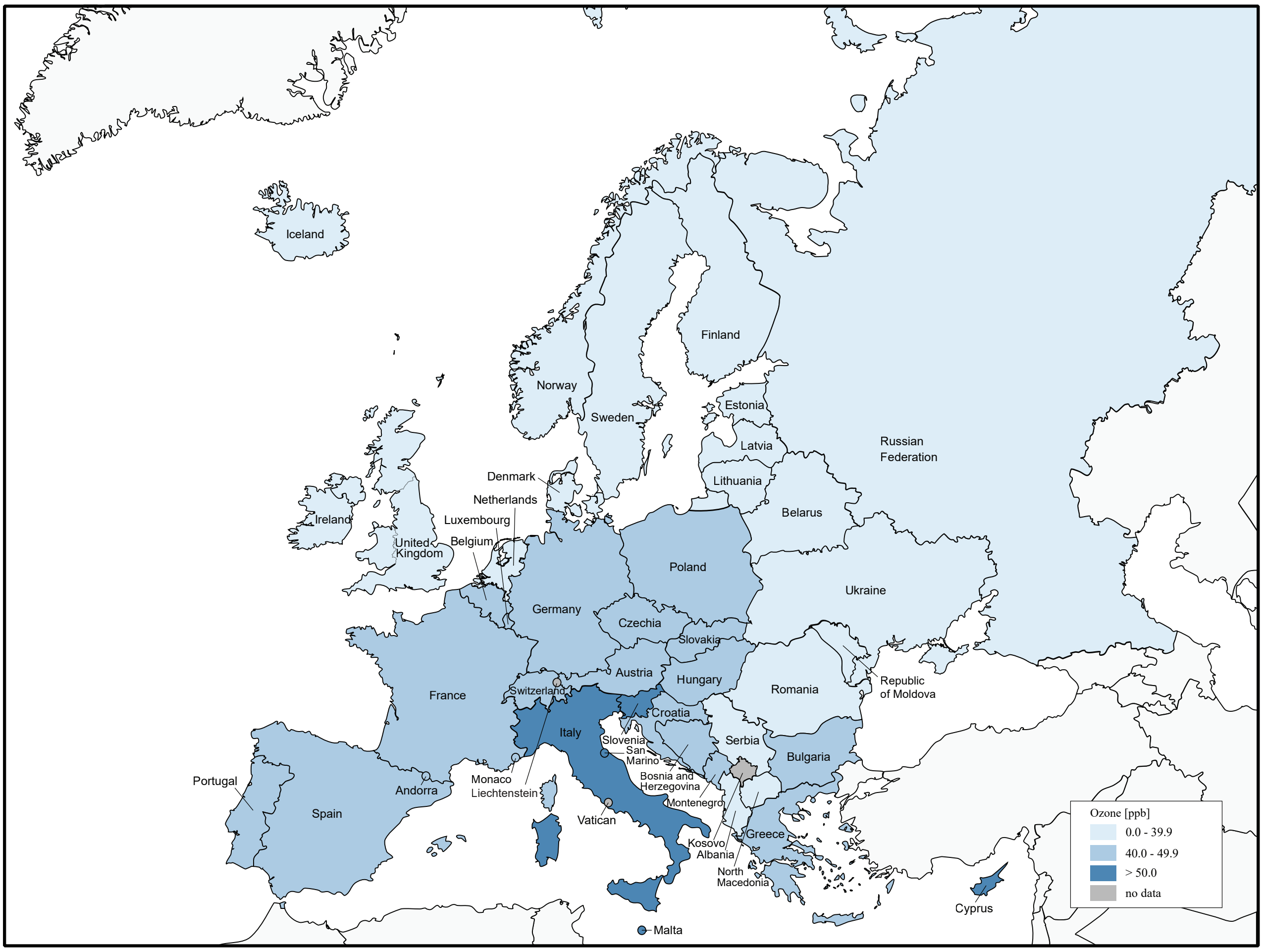

Supplementary Figure S3

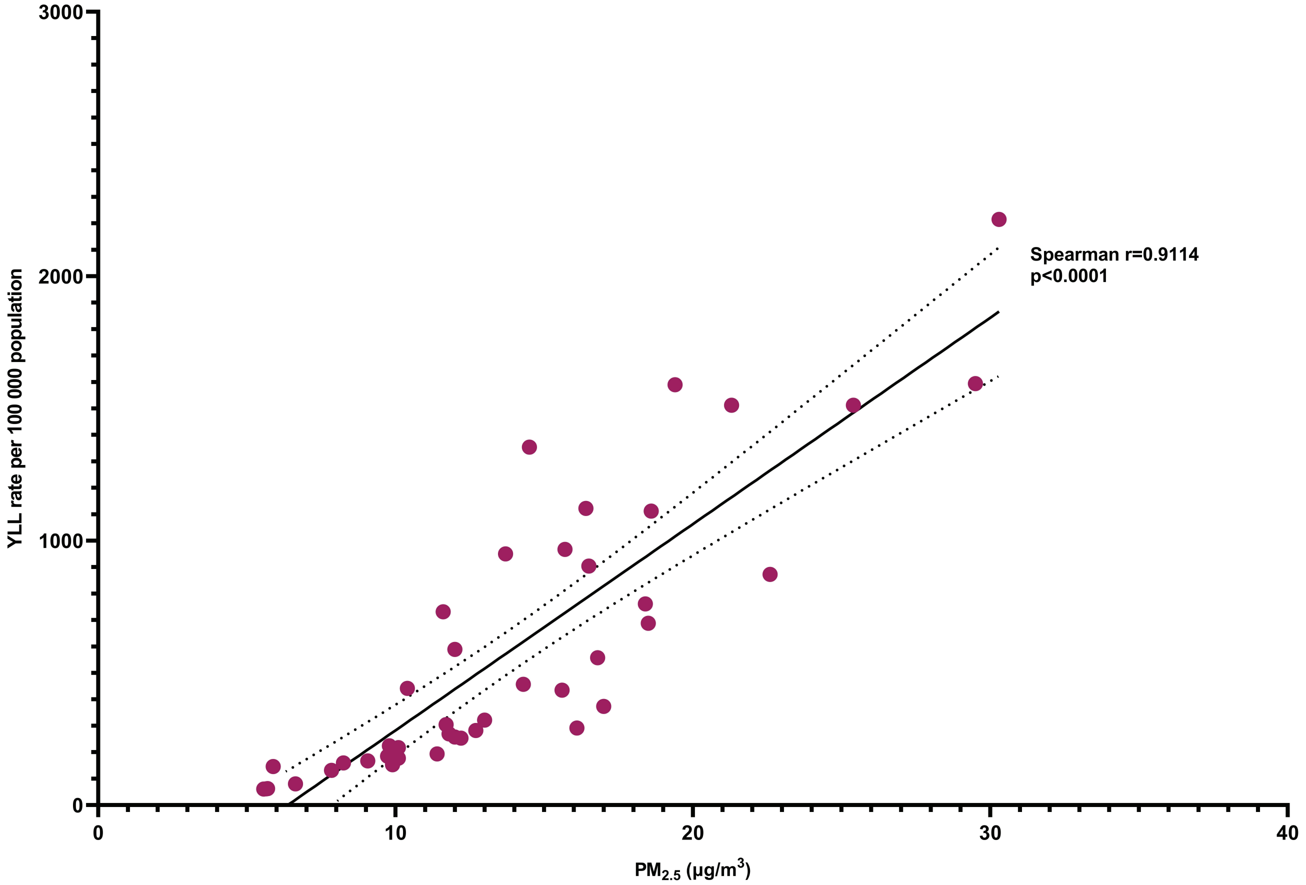

Supplementary Figure S4 (A)

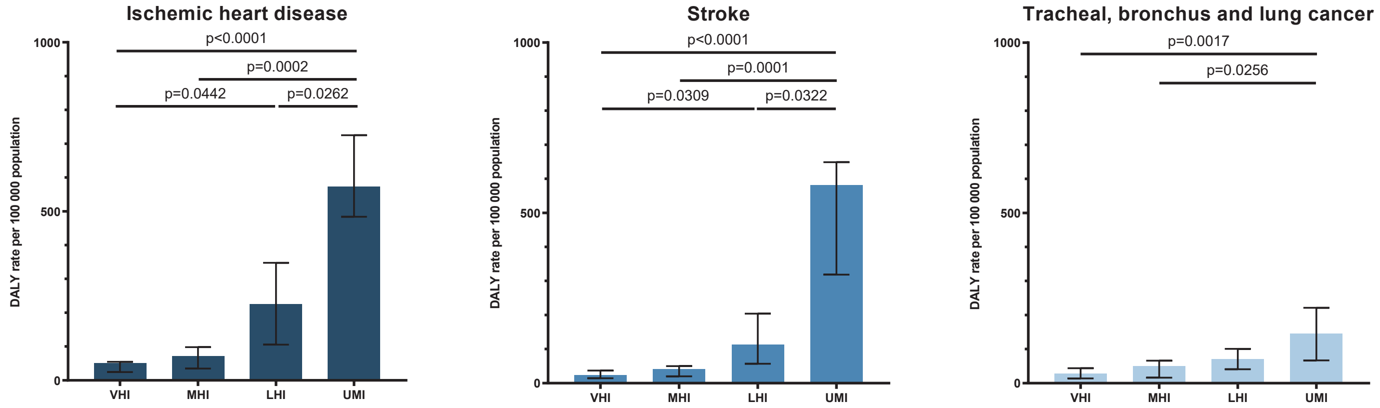

Supplementary Figure S4 (B)

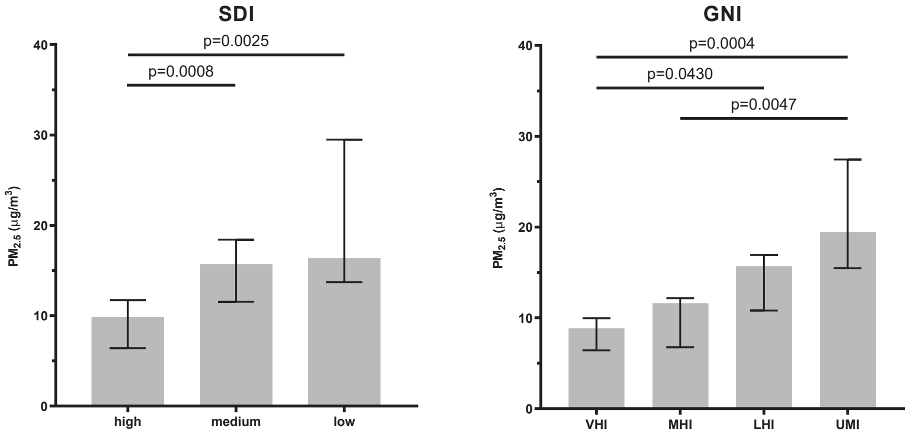

Supplementary Figure S5

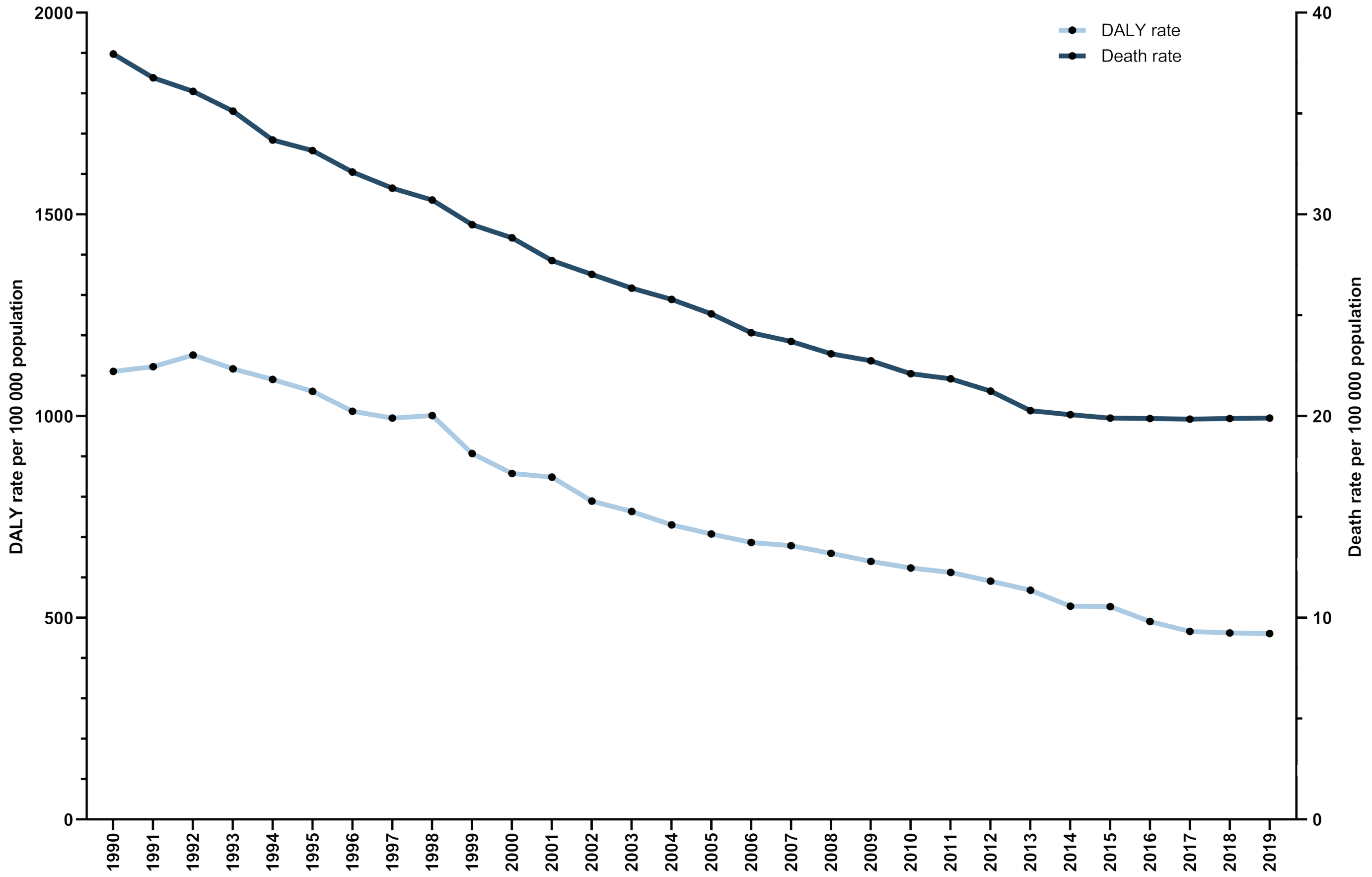

Supplementary Figure S6

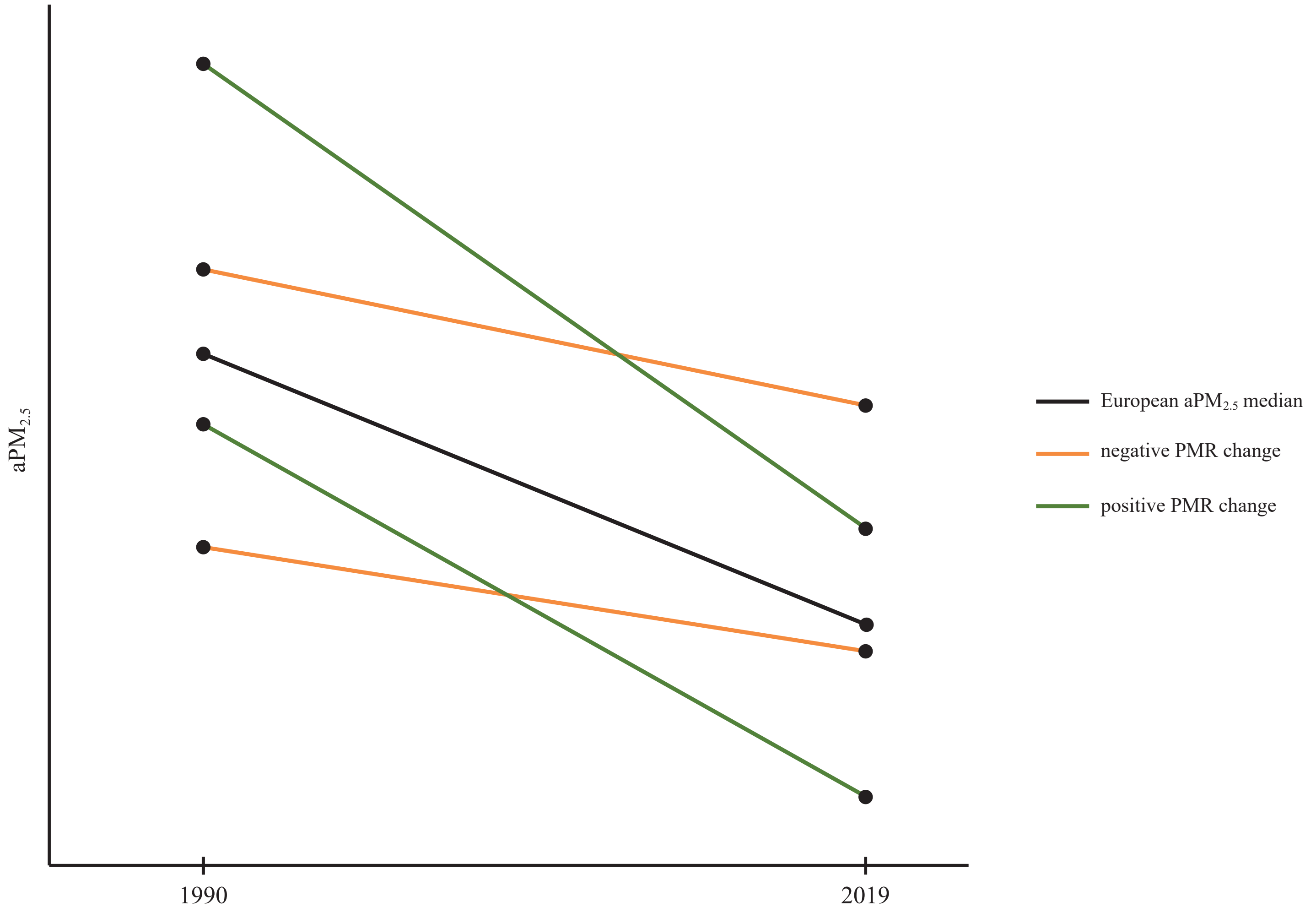

Supplementary Table S1

Country	1990	2019	Ambient $PM_{2.5}$ difference (%)
Albania	24.9	18.6	-25.3
Andorra	14.3	9.1	-36.6
Austria	21.3	12.2	-427
Belarus	25.2	16.4	-34.9
Belgium	21.1	12.7	-39.8
Bosnia and Herzegovina	36.7	29.5	-19.6
Bulgaria	28.0	19.4	-30.7
Croatia	26.1	18.5	-29.1
Cyprus	17.5	15.6	-10.9
Czechia	26.1	16.8	-35.6
Denmark	17.3	9.8	-43.4
Estonia	9.9	5.9	-40.6
Finland	8.8	5.6	-36.7
France	18.6	11.4	-38.7
Germany	20.6	11.8	-42.7
Greece	20.4	14.3	-29.9
Hungary	25.6	16.5	-35.6
Iceland	7.2	5.7	-21.1
Ireland	12.7	7.9	-38.2
Italy	26.9	16.1	-40.2
Latvia	20.9	12.0	-42.6
Lithuania	17.5	10.4	-40.6
Luxembourg	16.2	10.1	-37.7
Malta	16.5	13.0	-21.2
Monaco	10.2	11.7	14.7
Montenegro	33.1	21.3	-35.7
Netherlands	19.9	12.0	-39.7
North Macedonia	45.6	30.3	-33.6
Norway	11.8	6.6	-43.7
Poland	33.3	22.6	-32.1
Portugal	11.6	8.3	-28.9
Republic of Moldova	22.4	13.7	-38.8
Romania	22.1	15.7	-29.0
Russian Federation	18.8	11.6	-38.3
San Marino	12.6	9.9	-21.4
Serbia	35.8	25.4	-29.1
Slovakia	27.2	18.4	-32.4
Slovenia	26.6	17.0	-36.1
Spain	14.0	9.7	-30.5
Sweden	9.8	5.7	-42.2
Switzerland	18.0	9.9	-45.0
Ukraine	22.8	14.5	-36.4
United Kingdom	17.4	10.1	-42.0
EUROPE	20.4	12.2	-40.2
EUROPEAN UNION	20.4	12.2	-40.2

Supplementary Table S2

Country	Deaths (thousands)	Age-standardised death rate per 100,000 (UI)	Age-standardised DALY rate per 100,000 (UI)
Albania	2.3 (1.6 – 3.1)	54.9 (39.4 – 73.3)	1250.7 (910.8 – 1634.9)
Andorra	0.0 (0.0 – 0.0)	9.1 (5.2 – 13.9)	209.6 (120.7 – 311.6)
Austria	2.7 (2.0 – 3.4)	13.5 (10.2 – 17.0)	317.6 (236.9 – 399.4)
Belarus	8.6 (6.0 – 11.8)	53.4 (37.2 – 73.6)	1217.2 (847.4 – 1669.5)
Belgium	3.8 (2.8 – 4.8)	14.9 (11.1 – 18.8)	359.8 (269.1 – 452.9)
Bosnia and Herzegovina	4.8 (3.8 – 6.2)	84.4 (65.9 – 107.8)	1883.4 (1489.4 – 2375.7)
Bulgaria	11.0 (8.4 – 14.0)	76.1 (57.9 – 96.9)	1777.6 (1348.4 – 2249.6)
Croatia	3.4 (2.5 – 4.3)	36.9 (27.8 – 47.4)	842.6 (639.8 – 1071.5)
Cyprus	0.5 (0.4 – 0.6)	26.1 (20.0 – 33.2)	535.3 (413.3 – 673.0)
Czechia	6.6 (5.0 – 8.4)	30.4 (23.1 – 38.6)	738.3 (566.2 – 937.1)
Denmark	1.5 (0.9 – 2.1)	12.0 (7.7 – 16.7)	269.9 (171.5 – 369.6)
Estonia	0.2 (0.1 - 0.4)	7.3 (2.5 – 13.7)	178.0 (65.8 – 320.3)
Finland	0.4 (0.1 – 0.8)	3.1 (0.8 – 6.2)	79.3 (20.2 – 154.3)
France	14.3 (10.4 – 18.6)	9.3 (6.8 – 12.0)	230.1 (166.2 – 298.1)
Germany	29.3 (21.6 – 37.5)	14.0 (10.4 – 17.8)	351.1 (258.6 – 449.5)
Greece	6.2 (4.7 – 7.7)	23.1 (17.8 – 28.3)	546.4 (423.9 – 672.6)
Hungary	8.6 (6.4 – 11.0)	43.8 (32.6 – 55.8)	1080.6 (811.6 – 1378.2)
Iceland	0.0 (0.0 – 0.0)	3.1 (0.9 – 6.0)	79.1 (22.4 – 148.5)
Ireland	0.6 (0.3 – 0.8)	7.2 (3.8 – 11.0)	165.8 (86.6 – 250.4)
Italy	27.9 (21.9 – 33.9)	16.5 (13.1 – 20.0)	380.6 (304.4 – 462.9)
Latvia	1.2 (0.8 – 1.7)	29.1 (20.1 – 41.1)	674.1 (465.6 – 931.7)
Lithuania	1.3 (0.9 – 1.9)	21.8 (14.1 – 31.0)	502.0 (327.8 – 706.5)
Luxembourg	0.1 (0.1 – 0.1)	9.6 (6.4 – 13.0)	243.1 (160.6 – 331.0)
Malta	0.2 (0.1 – 0.2)	16.7 (12.3 – 21.1)	403.3 (296.4 – 517.5)
Monaco	0.0 (0.0 – 0.0)	15.7 (9.5 – 22.5)	366.0 (222.3 – 529.7)
Montenegro	0.7 (0.6 – 0.9)	77.6 (58.9 – 98.0)	1728.8 (1324.0 – 2174.3)
Netherlands	5.0 (3.6 – 6.4)	13.9 (10.1 – 17.7)	323.5 (236.7 – 412.8)
North Macedonia	3.3 (2.6 – 4.2)	118.4 (94.6 – 147.5)	2501.9 (1981.9 – 3149.2)
Norway	0.5 (0.2 – 0.8)	4.4 (1.8 – 7.3)	107.8 (47.4 – 179.5)
Poland	31.1 (24.8 – 38.0)	43.4 (34.5 – 53.2)	1057.9 (851.6 – 1284.9)
Portugal	2.4 (1.4 – 3.5)	8.8 (5.3 – 12.8)	206.6 (123.9 – 297.7)
Republic of Moldova	2.4 (1.4 – 3.4)	42.6 (25.7 – 60.0)	1055.7 (654.8 – 1474.6)
Romania	17.1 (12.8 – 22.5)	45.3 (34.1 – 59.4)	1098.1 (832.1 – 1410.2)
Russian Federation	77.5 (45.1 – 111.2)	33.4 (19.4 – 47.8)	799.6 (467.5 – 1140.8)
San Marino	0.0 (0.0 – 0.0)	9.2 (4.2 – 15.8)	226.8 (107.1 – 378.4)
Serbia	12.7 (9.9 – 16.1)	82.9 (65.3 – 105.0)	1762.1 (1385.7 – 2222.6)
Slovakia	3.6 (2.7 – 4.6)	39.4 (29.4 – 50.7)	894.9 (668.0 – 1146.4)
Slovenia	0.9 (0.7 – 1.3)	19.9 (14.4 – 27.0)	492.2 (366.1 – 642.8)
Spain	11.8 (8.0 – 15.8)	10.4 (7.2 – 13.8)	245.0 (168.4 – 329.4)
Sweden	0.8 (0.2 – 1.4)	3.2 (1.0 – 6.1)	79.2 (25.9 – 146.0)
Switzerland	1.6 (1.1 – 2.2)	8.2 (5.7 – 10.8)	198.4 (132.8 – 271.0)
Ukraine	46.1 (29.1 – 65.4)	60.9 (38.5 – 86.3)	1451.7 (908.9 – 2049.4)
United Kingdom	15.0 (10.0 – 20.4)	11.2 (7.5 – 15.2)	286.6 (195.9 – 386.6)
EUROPEAN UNION	192.4	16.5 (19.8)	380.6 (462.1)
EUROPE	368.0	16.7 (33.5)	403.4 (820.2)

Supplementary Table S3

Country	YLL rate attributable to air pollution			Death rate attributable to air pollution		
	YRR 1990	YRR 2019	YRR change (%)	DRR 1990	DRR 2019	DRR change (%)
Albania	3.71	3.46	6.74	2.93	3.29	-12.29
Andorra	0.43	0.52	-20.93	0.44	0.54	-22.73
Austria	0.89	0.79	11.24	0.89	0.81	8.99
Belarus	2.08	3.49	-67.79	1.88	3.2	-70.21
Belgium	0.94	0.88	6.38	0.93	0.89	4.3
Bosnia and Herzegovina	3.28	4.96	-51.22	2.99	5.05	-68.9
Bulgaria	3.29	4.95	-50.46	3.07	4.55	-48.21
Croatia	1.97	2.14	-8.63	1.89	2.21	-16.93
Cyprus	1.09	1.35	-23.85	1.25	1.56	-24.8
Czechia	2.14	1.73	19.16	2.02	1.82	9.9
Denmark	0.94	0.7	25.53	0.91	0.72	20.88
Estonia	1.3	0.45	65.38	1.18	0.44	62.71
Finland	0.42	0.19	54.76	0.39	0.19	51.28
France	0.57	0.6	-5.26	0.57	0.56	1.75
Germany	1	0.84	16	1	0.84	16
Greece	0.98	1.42	-44.9	1	1.38	-38
Hungary	2.72	2.81	-3.31	2.34	2.62	-11.97
Iceland	0.23	0.19	17.39	0.22	0.19	13.64
Ireland	0.77	0.41	46.75	0.78	0.43	44.87
Italy	0.98	0.91	7.14	0.98	0.99	-1.02
Latvia	2.01	1.83	8.96	1.83	1.74	4.92
Lithuania	1.48	1.37	7.43	1.36	1.31	3.68
Luxembourg	0.81	0.55	32.1	0.8	0.57	28.75
Malta	0.92	1	-8.7	0.93	1	-7.53
Monaco	0.37	0.95	-156.76	0.37	0.94	-154.05
Montenegro	2.43	4.71	-93.83	2.17	4.65	-114.29
Netherlands	0.9	0.8	11.11	0.88	0.83	5.68
North Macedonia	4.17	6.89	-65.23	3.67	7.09	-93.19
Norway	0.5	0.25	50	0.48	0.26	45.83
Poland	2.84	2.71	4.58	2.49	2.6	-4.42
Portugal	0.7	0.5	28.57	0.7	0.53	24.29
Republic of Moldova	3.54	2.96	16.38	3.13	2.55	18.53
Romania	2.98	3.01	-1.01	2.59	2.71	-4.63
Russian Federation	1.83	2.27	-24.04	1.63	2	-22.7
San Marino	0.33	0.55	-66.67	0.34	0.55	-61.76
Serbia	3.6	4.71	-30.83	3.28	4.96	-51.22
Slovakia	2.32	2.37	-2.16	2.09	2.36	-12.92
Slovenia	1.42	1.16	18.31	1.38	1.19	13.77
Spain	0.61	0.58	4.92	0.62	0.62	0
Sweden	0.35	0.19	45.71	0.35	0.19	45.71
Switzerland	0.68	0.47	30.88	0.69	0.49	28.99
Ukraine	2.1	4.21	-100.48	1.98	3.65	-84.34
United Kingdom	0.94	0.68	27.66	0.92	0.67	27.17

Ratio:

<0.50   0.50 – 0.99   1.00 – 1.49   1.50 – 1.99   2.00 – 2.49   ≥2.50

Supplementary Table S4

Country	GNI per capita	GNI category	SDI	SDI category
Albania	5240	UMI	0.681	low
Andorra	*No data*		0.894	high
Austria	51300	MHI	0.849	medium
Belarus	6280	UMI	0.745	low
Belgium	47350	MHI	0.851	high
Bosnia and Herzegovina	6150	UMI	0.718	low
Bulgaria	9410	UMI	0.764	medium
Croatia	14910	LHI	0.794	medium
Cyprus	27710	LHI	0.841	medium
Czechia	22000	LHI	0.828	medium
Denmark	63240	VHI	0.89	high
Estonia	23220	LHI	0.835	medium
Finland	49580	MHI	0.856	high
France	42400	MHI	0.834	medium
Germany	48520	MHI	0.898	high
Greece	20320	LHI	0.794	medium
Hungary	16140	LHI	0.791	medium
Iceland	72850	VHI	0.869	high
Ireland	62210	VHI	0.867	high
Italy	34460	LHI	0.801	medium
Latvia	17730	LHI	0.82	medium
Lithuania	18990	LHI	0.843	medium
Luxembourg	73910	VHI	0.895	high
Malta	27290	LHI	0.801	medium
Monaco	*No data*		0.902	high
Montenegro	9010	UMI	0.791	medium
Netherlands	53200	MHI	0.883	high
North Macedonia	5910	UMI	0.744	low
Norway	82500	VHI	0.913	high
Poland	15200	LHI	0.802	medium
Portugal	23080	LHI	0.743	low
Republic of Moldova	*No data*		0.696	low
Romania	12630	LHI	0.76	medium
Russian Federation	11260	UMI	0.805	medium
San Marino	*No data*		0.884	high
Serbia	7020	UMI	0.767	medium
Slovakia	19320	LHI	0.812	medium
Slovenia	25750	LHI	0.84	medium
Spain	30390	LHI	0.767	medium
Sweden	55840	MHI	0.872	high
Switzerland	85500	VHI	0.929	high
Ukraine	3370	UMI	0.736	low
United Kingdom	42370	MHI	0.847	medium

SUPPLEMENTARY LEGENDS

Supplementary Figure S1: Median European $aPM_{2.5}$ concentration from 1990 to 2019. The figure was made in Adobe Illustrator (version 24.1., URL: https://www.adobe.com/products/illustrator.html).

Supplementary Figure S2. Average seasonal population-weighted ozone concentrations in 1990 (a) and 2019 (b). Ppb – parts per billion. The figure was made in Adobe Illustrator (version 24.1., URL: https://www.adobe.com/products/illustrator.html).

Supplementary Figure S3. Correlation between YLL and $aPM_{2.5}$ concentration in 2019.

Supplementary Figure S4. (a) Comparison of countries by GNI per capita and DALY rate attributable to air pollution for IHD, stroke and TBL cancer in 2019. Countries are categorised by income level into 4 categories: very high income (VHI), moderate high income (MHI), lower high income (LHI), and upper middle-income (UMI). Groups are represented as median with interquartile range (IQR). (b) Comparison of countries based on GNI per capita, SDI and $aPM_{2.5}$ concentration in 2019.
Groups are represented as median with interquartile range (IQR). Statistical analysis by Kruskal-Wallis test by ranks with Dunn's multiple comparisons test. Bonferroni correction for multiple tests was used to adjust significance values.

Supplementary Figure S5. DALY rate and death rate in Europe from 1990 to 2019.

Supplementary Figure S6. Visualization of PMR change calculation for the period between 1990 and 2019. This figure is a hypothetical model that does not represent true values for any country, but serves as a guide to better understand and graphically visualize our calculation. The figure shows 4 possible hypothetical outcomes of our calculation where countries represented by the orange and green lines all improved their $aPM_{2.5}$ in 2019 compared to 1990, but only the countries represented by the green lines improved it more than the European median (black line). Thus, these latter countries are represented by a positive PMR change value. More specifically, green lines have a slope that is at least as steep as the slope of the European median line, indicating that their reduction of $aPM_{2.5}$ is at least as big as the European median reduction. The opposite is valid for orange lines. Another hypothetical situation is highlighted in the figure too. Both lines (orange and green) which are under the European median had better $aPM_{2.5}$ in 1990 and 2019, but the countries represented by the orange line did not manage to keep the slope of reduction as steep as the European median. Because of this, these countries would be represented by a negative PMR change value, indicating that they cannot follow the trend of reduction of the European $aPM_{2.5}$ median. On the other hand, the green line under the European median was better both in 1990 and 2019 than the European median, but managed to steepen its slope even more than Europe, and thus, these countries would be represented by a positive PMR change value, indicating they have managed to further increase their reduction, even more than Europe overall. The same analogy can be applied to DARR, DRR, and YRR changes.

Supplementary Table S1. The average annual population-weighted $aPM_{2.5}$ concentration in European countries for 1990 and 2019.

Supplementary Table S2. Deaths, age-standardised death rates and DALY rate attributable to air pollution for European countries in 2019.

Supplementary Table S3. Ratio of YLL rate attributable to air pollution and death rate attributable to air pollution in 1990 and 2019 for European countries.

Supplementary Table S4. Categorization of European countries by SDI and GNI in 2019. GNI is expressed in US dollars.
